# AGC family kinase of *Entamoeba histolytica*: Decoding the members biochemically

**DOI:** 10.1371/journal.ppat.1012729

**Published:** 2024-11-19

**Authors:** Azhar Ahmad, Vikas Kumar, Tushar Kushwaha, Akash Kumar, Deepak Sehgal, Krishna K. Inampudi, Somlata

**Affiliations:** 1 Multidisciplinary Centre for Advanced Research and Studies, Jamia Millia Islamia, New Delhi, India; 2 Department of Biophysics, All India Institute of Medical Sciences, New Delhi, India; 3 Department of Life Sciences, School of Natural Sciences, Shiv Nadar University, Greater Noida, Uttar Pradesh, India; University of California Davis, UNITED STATES OF AMERICA

## Abstract

*Entamoeba histolytica*, a protozoan parasite, is the causative agent of amoebiasis, which is a significant global health concern. The virulence mechanisms underlying its pathogenicity are multifaceted and complex. However, endocytic processes and motility are well accepted virulence determinants. As previously reported, an AGCK family kinase, EhAGCK1 to be involved in trogocytosis exclusively while another one from same family named EhAGCK2 participates in all actin dependent endocytic processes. As the kinase dead mutants of EhAGCK1 showed significant defect in destruction of live host cells and also the localisation pattern of same is distinguishable from EhAGCK2. From observations so far, it appears that former initiates a distinguishable signaling cascade. In this work, we have demonstrated distinct biochemical properties of kinases involved in related yet distinguishable endocytic processes for the first time. Our biochemical characterization highlights distinct ion dependency of EhAGCK1 along with substrate specificity. We also show upstream activator of these kinases, 3-phosphoinositide dependent kinase 1 (PDK1) activity and its role in activating the kinase activity. The kinases exhibit property of autophosphorylation, and which may regulate the kinase activity subsequently. Summarily, these studies show that EhAGCK1 and EhAGCK2 show distinct biochemical properties which further confirm their unique role in related endocytic processes of trogocytosis and phagocytosis.

## Author summary

The protozoan parasite *E*. *histolytica* causes amebiasis in humans. The trophozoites depend on variety of endocytic processes to survive and cause pathogenesis in the gut of host system. Phagocytosis and trogocytosis are two related endocytic processes known to contribute to the pathogenesis by the parasite in vivo. The two processes are phenotypically different, trogocytosis being fast paced resembling nibbling and specific to live host cells. In terms of molecular mechanism of these processes, an AGC family kinase, EhAGCK1 is known to be involved in trogocytosis specifically while another one EhAGCK2 is involved in all types of endocytic processes including fluid uptake. Our biochemical characterization of these two kinases reveal that they have distinct substrate specificity and ion requirement. The overall biochemical assessment indicates the two kinases belonging to same family are involved in the phagocytic and trogocytic pathway distinctly. Hence, although the phagocytosis and trogocytosis at molecular level appear to be related but they also involve steps unique to each pathway.

## Introduction

*Entamoeba histolytica*, a protozoan parasite responsible for amebiasis, imposes a significant global health burden particularly in developing nations. Annually, approximately 50 million individuals contract the infection, leading to an alarming 100,000 deaths [1]. This parasite’s phagocytic and motility abilities are well-recognized behaviours, serving as crucial virulence determinants [2]. Until recently, it was believed that *E*. *histolytica* exclusively caused host cell death through contact-dependent mechanisms, followed by the phagocytosis of the deceased cells [3]. However, another process by which parasite invades host intestinal tissues is by trogocytosis, which was reported in 2014. During trogocytosis, the amoeba nibbles the live host cells in small bites, resulting in compromised plasma membrane integrity, elevated cytosolic calcium levels and eventual cell death [4]. Although, trogocytosis occur specifically when trophozoites comes in contact with live host cells yet it shares similarities with phagocytosis at molecular level, implicating molecules like EhC2PK, Phosphoinositide 3-Kinase(PI3K) and actin [4].

*E*. *histolytica* interaction with host cells initiates a cascade of kinases and phosphatase activation, modifying phosphoinositides in the amoeba’s plasma membrane [5]. These phosphoinositides regulate essential cellular functions like cytoskeleton reorganization and membrane trafficking [6]. Also, various receptors on the amoebic cell surface, including Gal/GalNAc lectin [7], TMK96 [8], TMK39 [9], SREHP [10], and EhROM1[11] play a crucial role in the parasite’s interaction with human host cells. The interaction between an amoebic cell and target host cells induces changes in the composition of phosphoinositides within the parasite’s plasma membrane [12]. This alteration results from the activation of Phosphatidylinositol Phosphate Kinase (PIPK), which leads to the generation of two key phosphoinositides: Phosphatidylinositol 4,5-bisphosphate (PtdIns (4,5)P2) and Phosphatidylinositol 3,4,5-trisphosphate (PtdIns (3,4,5)P3) [12]. Subsequently, these changes in phosphoinositides trigger the recruitment of downstream AGC family kinases [13]. The AGC kinases belong to a subgroup of serine/threonine protein kinases, with notable members including Protein Kinase A (PKA), Protein Kinase G (PKG), and Protein Kinase C (PKC) [13]. The genome of *E*. *histolytica* encodes a total of 24 AGC family kinases, but so far, only two have been confirmed to play a role in the endocytic processes [13]. AGC kinases are known to undergo phosphorylation at two highly conserved regulatory motifs, namely the T/activation loop and the hydrophobic motif to achieve maximal activation. These motifs are located within the catalytic domain and in a non-catalytic region following the kinase domain, respectively. Additionally, AGC kinases possess a crucial phosphorylation site referred to as the ’turn motif,’ which is responsible for their integrity and activation [14].

Although it is established that the PI3K-PKC activity is required for host cell killing by *E*. *histolytica*, the detailed mechanism and the specific proteins involved in this pathway remain unidentified [15]. In 2017, it was reported that EhAGCK1, a member of AGC family kinase is exclusively involved in trogocytosis. This represents the first instance of a kinase specifically associated with this process [13]. EhAGCK1 has a distinct protein structure comprising a Pleckstrin Homology (PH) domain at the N-terminal, enabling it to bind Phosphatidylinositol 3,4,5-trisphosphate (PtdIns (3,4,5) P3). Following the PH domain is the kinase domain, with AGC motif at C-terminal. EhAGCK1 decorates the plasma membrane at the trogocytosis site and dissociates from the membrane at the site as soon as the host cell fragment is ingested by the trophozoites [13]. On the other hand, another AGC family kinase, EhAGCK2, which shares 51% sequence identity with EhAGCK1, is involved in various actin-dependent endocytic processes, including phagocytosis, trogocytosis, and pinocytosis [13]. Like EhAGCK1, EhAGCK2 possesses a PH domain at the N-terminal which interacts with PtdIns (3,4,5) P3, followed by the kinase domain. Additionally, EhAGCK2 has an AGC kinase motif at the N-terminal. Both kinases are transiently present at endocytosis sites, leaving once the process is complete, making them undetectable in phagosome proteome data reported earlier [13].

However, in spite of confirmed role in phagocytosis and trogocytosis the cascades initiated by these AGC family kinases remains to be explored. The information about upstream activator and downstream substrates for both EhAGCK1 and EhAGCK2 will be useful in understanding their role in the endocytic processes and hence possibly link them to actin dynamics and membrane remodelling occurring during the fast-paced trogocytosis and relatively slow-paced phagocytosis events. From previous studies, it is evident that sequentially EhAGCK1 and EhAGCK2 are similar to human Akt kinases [13], which are further dependent on PDK1 (3-phosphoinositide dependent kinases 1) for their activation. We also try to determine the human homologue of PDK1 in *E*. *histolytica* responsible for phosphorylating EhAGCK1 and EhAGCK2 and further activating their kinase activity. In this report, we present the first comprehensive biochemical characterization of AGC family kinase specifically EhAGCK1 and EhAGCK2 by creating various mutants of the kinases and wild type recombinant proteins. The observations from *in vitro* kinase assay with synthetic peptides from human and *E*. *histolytica* origin have indicated of substrate specificity implying involvement in pathways of distinct endocytic events. This study has helped to initiate and recognise the studies pertaining to dissecting out the cross talking endocytic pathways in protozoan parasite *E*. *histolytica* and understand the processes contributing to pathogenesis with more clarity.

## Results

### Structural comparison of EhAGC kinases with human Akt1

**Fig 1:**
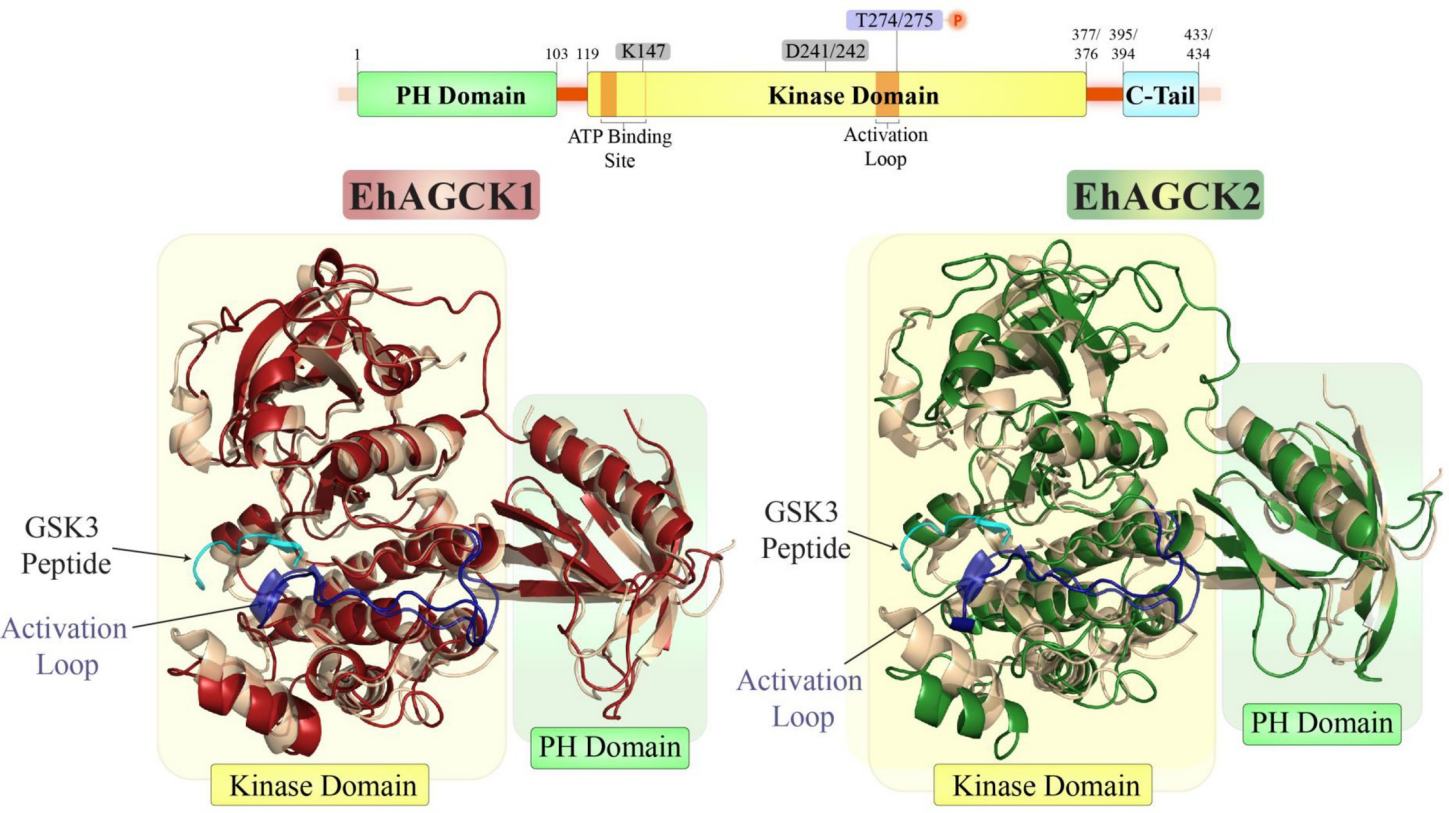
Structural comparison of EhAGCK1 and EhAGCK2 with human Akt1. The structures of EhAGCK1(EHI_188930) and EhAGCK2(EHI_053040) were modelled using AlphaFold3 [49] and refined by 200 ns molecular dynamics simulations using GROMACS [50]. Both EhAGCK1 and EhAGCK2 were stable throughout the 200 ns simulations. The MD refined structures were structurally aligned with the PH and kinase domains of human Akt1 (PDB ID: 3OCB and 6HHG) [51,56] using TM-align [52]. The kinase and the PH domains of EhAGCK1 and EhAGCK2 showed high structural similarity to the human Akt1 kinase with TM scores >0.75 (TM score of 1 indicates perfect match). The schematics of EhAGCK1 and EhAGCK2 genes show different domains of the kinases along with ATP binding site and activation loop. The phosphorylation site of EhAGCK1/EhAGCK2 (T274/275) is also highlighted along with the residues K147 and D241/242 which were mutated to make the kinase-dead variants of EhAGCK1 and EhAGCK2. The structures of EhAGCK1 (red), EhAGCK2 (Green) are shown in cartoon representation overlayed on the crystal structure of human Akt1 (yellow) bound to GSK3 peptide (shown in cyan) (PDB ID: 3OCB and 6HHG). The activation loop is shown in purple.

Our *in-silico* analysis, led to the conclusion that EhAGCK1 and EhAGCK2 exhibit homology with human Akt isoforms in their conserved domains and residues. EhAGCK1 displays a remarkable 96% similarity in its kinase domain when compared to Akt3, while EhAGCK2 demonstrates a significant 94% similarity in its kinase domain with Akt2 as shown in **S1 Fig** [13]. Akt isoforms bind to PtdIns(3,4,5)P3 and PtdIns(3,4)P2 in the cell membrane, which is generated by PI3K in response to stimuli like insulin and growth factors. **Fig 1** presents a structural comparison of EhAGCK1 and EhAGCK2 with the human Akt1 isoform, showing a high level of structural similarity among the kinases. The Akt1 kinase is composed of four distinct domains: an N-terminal PH domain, an interdomain linker, a kinase domain, and a C-terminal regulatory tail. While the PH domain and the kinase domain exhibits significant structural similarity, variations were observed in the interdomain linker and C-terminal regulatory tail. The models of EhAGCK1 and EhAGCK2 kinase domains resemble the typical catalytic domain fold of kinases of AGC family having a small N-lobe and a larger C-lobe, with high structural similarity to human Akt1 showing an RMSD of 0.95Å and 1.3Å respectively. The ATP binding site harbouring the G loop is well-conserved in sequence and structure. The multiple alignment shows presence of asparagine in the activation loop which may lead to polar and rigid conformation in EhAGC kinases. The length and rigidity of the activation loop can have implications for the stringency of phosphorylation and cofactor binding [16]. However, the essential residues within the kinase domain were observed to be conserved, including both catalytic and phosphorylated residues, the DFG motif, and R273 within the catalytic loop, which safeguards Thr308 from dephosphorylation [17].

The PH domain also shows sequence and structure similarity in the lipid-binding pocket as the residues that bind to phosphate groups of phospholipids were found to be conserved in both EhAGCK1 and EhAGCK2. The β1-β2 loop in the PH domain, however, is more flexible in EhAGC kinases as they have glycine in place of the sentry Glu17 which is crucial for targeting PtdIns(3,4,5)P3, yet both EhAGCK1 and EhAGCK2 bind PtdIns(3,4)P2 and PtdIns(3,4,5)P3 as shown previously by Somlata et al [13]. Another comparable difference in EhAGCK1 and EhAGCK2 structure is the shorter interdomain linker connecting PH domain with the kinase domain as compared to human Akt1, limiting the flexibility of PH domain. Lastly, the disordered C-terminal regulatory region is shorter in EhAGC kinases than human Akt1. EhAGCK1 and EhAGCK2 kinases have Thr430, homologous to Ser473, phosphorylation of which by mTORC2 or DNA-PK is critical for activation and regulation of human Akt1 kinase [18] but lacks the homologous residues of Ser477 and Thr479 from human Akt1, which are phosphorylated by Cdk2/Cyclin-A in the nucleus [19]. This may be considered as reason for EhAGCK1 and EhAGCK2 to function in endocytic processes and not observed to be localised in nucleus.

### Identification of EhPDK1 as a putative activator of EhAGC kinases

PDK1 occurs widely throughout evolution, consisting of an N-terminal kinase domain followed by a linker region without a noticeable hydrophobic motif (HM) and a C-terminal PH domain [20]. PDK1 is a constitutively active enzyme and is considered a key mediator of PI3K pathway and therefore plays an important role in downstream growth factor [20]. Based on their higher similarity to Akt isoforms, it’s possible that EhAGCK1 and EhAGCK2 could be phosphorylated by proteins similar to PDK1, making these proteins potential upstream activators for *E*.*histolytica* AGC protein kinase. As mentioned earlier, we observed a remarkable similarity between EhAGCK1, EhAGCK2, and human PKB/Akt along with its isoforms. Human Akt protein and its isoforms undergo phosphorylation at the activation loop (T308 in Akt1) by human PDK1 protein [21]. Given this significant resemblance, it is plausible to hypothesize that PDK1-like gene to exist in *E*. *histolytica* genome. To investigate this, we performed a protein blast using the human PDK1 protein as the query sequence against the *E*. *histolytica* genome, aiming to identify EhPDK1 protein candidates.

**Fig 2:**
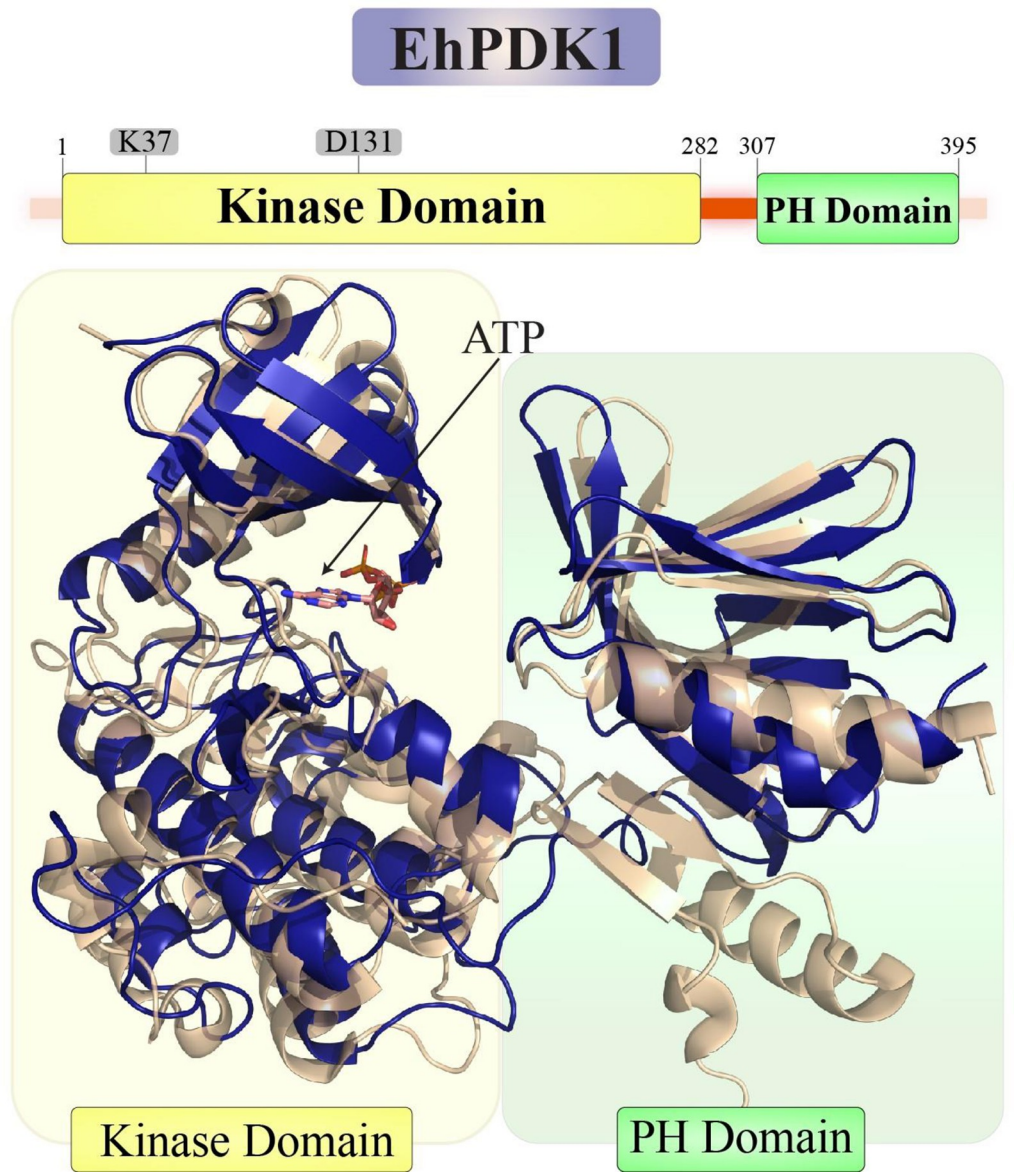
Structural Comparison of EhPDK1 with human PDK1. The structure of EhPDK1(EHI_095940) was modeled using AlphaFold3 and refined by 200 ns molecular dynamics simulations similar to EhAGCK1 and EhAGCK2. EhPDK1 was stable throughout the 200 ns trajectory. The MD refined structures were structurally aligned with the crystal structure of human PDK1 (PDB ID: 3HRF) [53] using TM-align. EhPDK1 showed high structural similarity to the human PDK1 with TM scores 0.86 (TM score of 1 indicates perfect match). The schematics of the EhPDK1 gene delineate various domains of the kinase, including the N-terminal kinase domain and the C-terminal PH domain, as well as the ATP binding site. Mutations were introduced at residues K37 and D131 to create kinase-dead variants of EhPDK1. The structure of EhPDK1 (Blue) is shown in cartoon representation overlayed on the crystal structure of human PDK1 bound to ATP (PDB ID: 3HRF) shown in yellow.

The Protein Blast results yielded intriguing findings, indicating that *E*. *histolytica* possesses two PDK1-like proteins with accession numbers EHI_095940 (EhPDK1) and EHI_006200 (EhPDK2). Both *E*. *histolytica* PDK1 proteins share 66% query coverage and 100% sequence Identity. However, analysis revealed that the EhPDK2 protein with accession number EHI_006200 lacks the critical conserved motif and residues essential for kinase activity, indicating the possibility of being a pseudo kinase as observed by sequence alignment (**S2A Fig)**. In contrast, the EhPDK1 protein with accession number EHI_095940 possesses the necessary motif and residues for kinase activity shown in **S2B Fig**. Therefore, for further studies, we have selected the EhPDK1 (EHI_095940). Subsequently, we performed multiple sequence alignments between EhPDK1 and human PDK1 protein sequences. The structure of EhPDK1 was modelled and compared with the structure of human PDK1 (**Fig 2**). Together, these results revealed that the ATP binding region and catalytic site responsible for phosphorylation are conserved between the *E*. *histolytica* and human PDK1 proteins, as shown in **S2C Fig**.

### Expression and purification of recombinant wild and mutant proteins

EhAGCK1 and EhAGCK2 proteins, including wild-type, kinase-dead, and constitutively active variants, were expressed in *E*. *coli* C41(DE3) cells and subsequently purified using Ni^2+^-NTA resin. The molecular masses of all variants of EhAGCK1 and EhAGCK2 proteins were determined to be around 50 kDa. Similarly, for EhPDK1, both wild-type and kinase-dead proteins were expressed in *E*. *coli* BL21(DE3) cells and purified using GST-sepharose resin. The molecular mass of EhPDK1 protein was found to be 68.7 kDa. The protein expression profiles of EhAGCK1, EhAGCK2, and EhPDK1 are shown in **S3A–S3C Fig** respectively. These purified proteins were utilized for the biochemical characterization studies.

### Assessing the kinase activity of the AGC kinases in the presence of human GSK3 peptide

**Fig 3:**
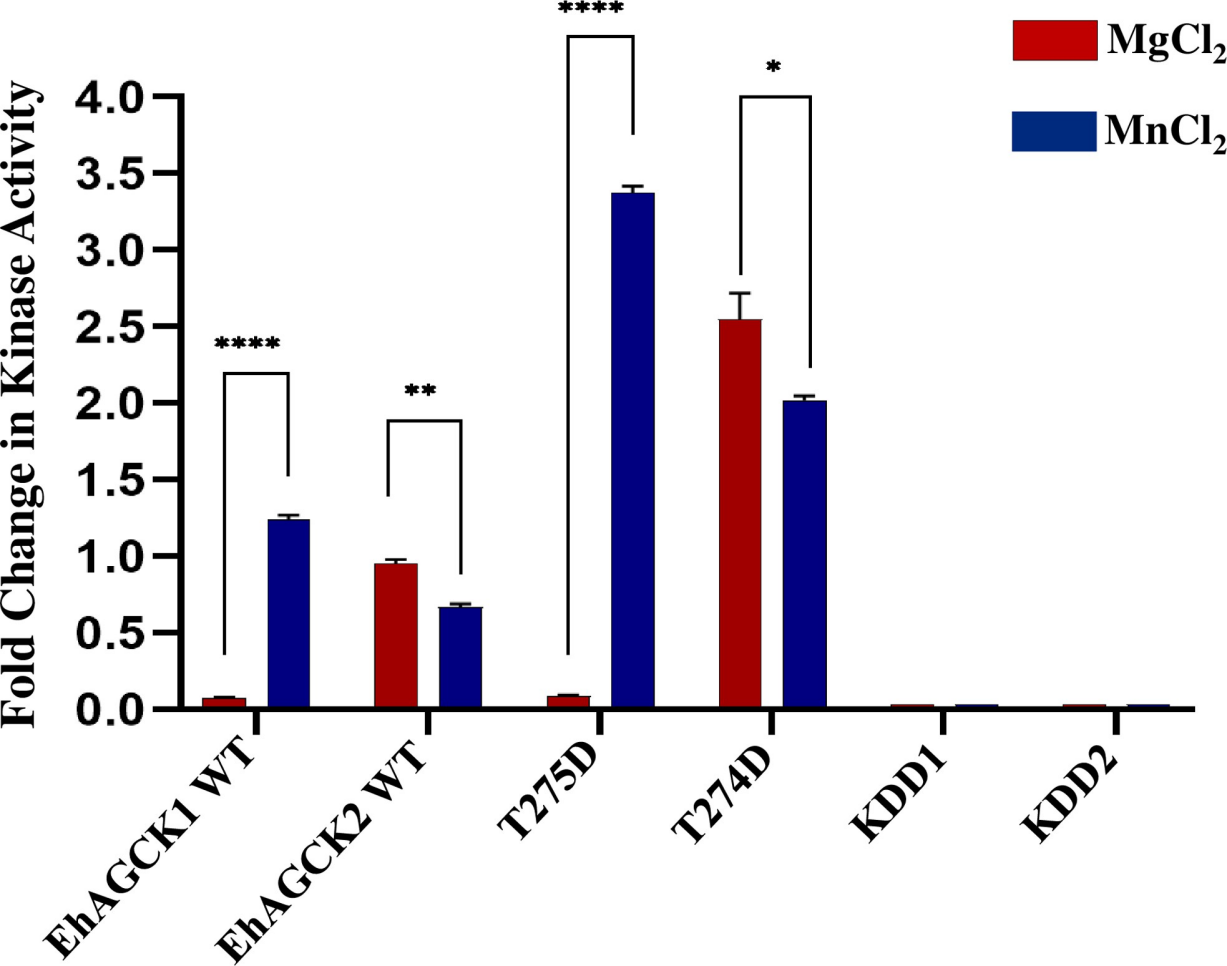
Optimization of ions for kinase activity of EhAGCK1 and EhAGCK2 in the presence of human GSK3 peptide substrate. Full-length recombinant purified wild-type proteins (EhAGCK1 WT and EhAGCK2 WT), constitutively active variants (T275D and T274D), and kinase-dead mutants (KDD1 and KDD2) were subjected to an assay with 50 μM HsGSK3 peptide substrate and 250 μM ATP for 60 minutes at 30°C in the presence of MgCl_2_ and MnCl_2_ buffer. The results, as illustrated in the graphs, show the fold change in kinase activity for the wild-type and variant proteins. Specifically, the assay indicated that wild-type EhAGCK1 is only active in the presence of MnCl_2_ buffer, while EhAGCK2 phosphorylates the peptide substrates in both MgCl_2_ and MnCl_2_ buffers. Furthermore, the fold change in kinase activity for the constitutively active variants (T275D and T274D) in the presence of the HsGSK3 peptide substrate is also represented, highlighting their differential activity profiles compared to the wild-type proteins. The X-axis displays the different protein variants, while the Y-axis shows the fold change in kinase activity. Error bars indicate the mean standard error. Values are derived from duplicate measurements across three independent experiments (n = 6). A Student’s t-test was employed to compare the activity of EhAGCK1 and EhAGCK2 protein variants in the presence of MgCl_2_ and MnCl_2_ buffers. Statistically significant differences were observed, ranging from *p-value ≤ 0.05, **p-value ≤ 0.01, ****p-value ≤ 0.0001.

The purified full-length recombinant EhAGCK1 and EhAGCK2 proteins were used for determining potential kinase activity using a non-radioactive luminescent based ADP-Glo kinase assay kit (Promega, USA), which measures ADP formation during the phosphate transfer reaction [22]. The screening included recombinant full-length wild-type proteins (EhAGCK1 WT and EhAGCK2 WT), constitutively active variants (T275D for EhAGCK1 and T274D for EhAGCK2), and kinase-dead domain (KDD) proteins KDD1(K147A and D241A) for EhAGCK1 and KDD2(K147A and D242A) for EhAGCK2. As previously mentioned, *in-silico* analysis of EhAGCK1 and EhAGCK2 revealed conserved domains and residues essential for kinase activity, similar to those found in human Akt kinases [13]. Therefore, human GSK3 (HsGSK3) peptide (GRPRTTSFAESCK), an endogenous substrate for Akt kinases, was used to assay kinase activity [23]. The kinase activity assays were performed using 50 μM peptide substrate, Mg^2+^, and various kinase concentrations from 0 μM to 80 μM, followed by addition of 250 μM ATP for reaction initiation. These results revealed that EhAGCK1 WT did not utilize the HsGSK3 peptide as a substrate **(Figs 3 and S4A)** while EhAGCK2 WT phosphorylates HsGSK3 peptide under same reaction conditions **(Figs 3 and S4B)**. Also, the kinase assay results indicate the residues predicted to be responsible for activation of kinases are indeed correct for EhAGCK2 WT, as phosphomimetic mutant (T274D) which is constitutively active shows kinase activity more than twice the wild type kinase *in vitro* **(Figs 3 and S4B)**. Hence, phosphorylation at T274 is important for activation of EhAGCK2 for optimal kinase activity.

### Activation of EhAGCK1 by Mn^2+^ ion

According to the literature and our past experience, many enzymatic proteins in parasites show enhanced activity in the presence of Mn^2+^ ion *in vitro* [24]. Moreover, the TORC1 complex, present in both yeast and mammals, distinguishes itself from the majority of serine/threonine kinases by exhibiting enzymatic activity in the presence of Mn^2+^ ion instead of the more common Mg^2+^ ion [25]. This specific divergence in cation preference underscores the unique characteristics of TORC1. Moreover, within the AGC kinase family, the kinases in *E*. *histolytica* can be classified into the subgroup of serine/threonine kinases [26]. Like many phosphotransferases, kinases require a divalent cation to coordinate the phosphate groups in the nucleotide triphosphate substrate and can be activated or inactivated by cation binding at an auxiliary site [25]. To determine *in vitro* cation preferences, we measured HsGSK3 peptide substrate phosphorylation in the presence of Mn^2+^ for EhAGCK1 WT, EhAGCK2 WT, and their constitutively active and dead enzymes. Interestingly, the results revealed that EhAGCK1 showed a significant increase in substrate phosphorylation activity in the presence of Mn^2+^ compared to EhAGCK2 (**Figs 3 and S4C**). The full length EhAGCK1 WT kinase showed a 16-fold increase in activity with HsGSK3 peptide as substrate, while the constitutively active mutant (T275D) exhibited an even higher increase, and the dead-kinase (KDD1) protein remained inactive **(Figs 3 and S4C)**. It should be noted that in the absence of Mn^2+^, EhAGCK1 could not accept HsGSK3 peptide as substrate, suggesting a role for the ion in substrate recognition. In contrast, EhAGCK2 WT and its cognate mutants showed no increment, in fact, a small decrease in the average kinase activity was observed in presence of Mn^2+^ (**Figs 3 and S4D**). Overall, the assay revealed the ion specificity between the two kinases may influence enzymatic activity and regulate the downstream. The fold change kinase activity between MgCl_2_ and MnCl_2_ for EhAGCK1 and EhAGCK2 have been summarised in **S1 Table**.

### Screening of *Entamoeba histolytica* peptides as a substrate for EhAGCK1 and EhAGCK2

**Fig 4:**
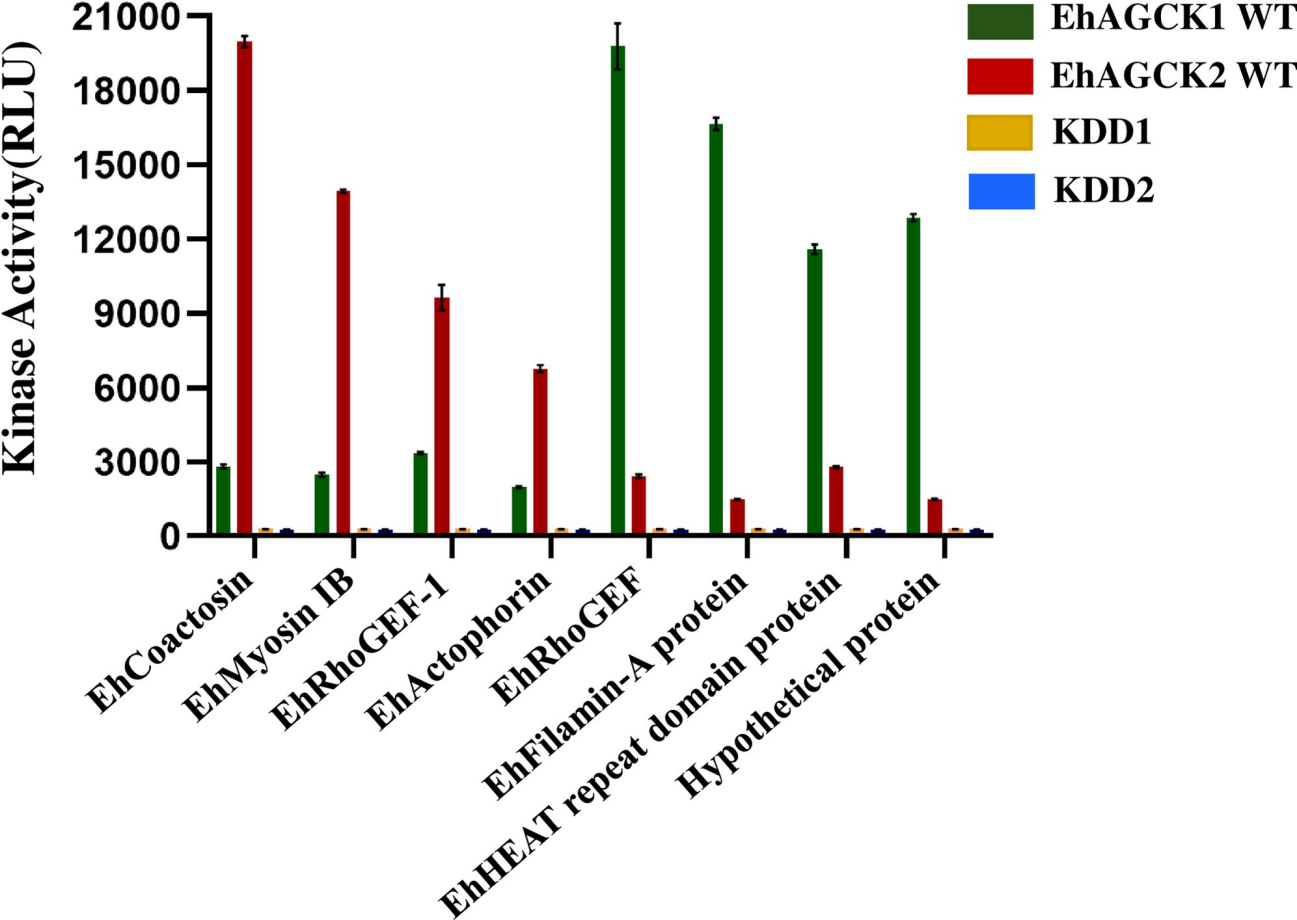
The bar graph shows the kinase activity of EhAGCK1 and EhAGCK2 in the presence of *E*. *histolytica* peptide substrates. The assay was performed in the presence of 20 μM of either EhAGCK1 WT or EhAGCK2 WT and kinase dead proteins as control, along with 250 μM ATP, and eight different peptide substrates, namely Coactosin (EHI_168340), Unconventional Myosin IB (EHI_110810), RhoGEF-1 (EHI_159500), Actophorin (EHI_197480), RhoGEF (EHI_008090), Filamin-A interacting protein (EHI_025370), HEAT repeat domain-containing protein (EHI_050150), and Hypothetical protein (EHI_025430) as marked in the figure. The results here are reported after 1 hr of kinase assay showing clear substrate specificity The X-axis displays the different peptide substrates, while the Y-axis shows kinase activity in terms of relative luminescence units (RLU). Error bars indicate the mean standard error. Relative luminescence unit (RLU) values are derived from duplicate measurements across three independent experiments (n = 6).

To perform subsequent experiments, we first established the optimal reaction conditions for EhAGCK1 and EhAGCK2 through rigorous *in vitro* kinase assays. However, their relevance in pathways leading to pathogenesis could be discerned by their *in vivo*, substrate identity. As the amoebic proteome comprises of 8000 proteins [26], identifying the specific substrate for a protein kinase is challenging task. To address this, we accessed the literature and thoroughly shortlisted proteins which are relevant to endocytic processes. Akt kinases, EhAGCK1 and EhAGCK2 are supposed to be downstream of PI3K, the article published by Jhingan et al provided the phosphoproteome in response to wortmannin treatment of the trophozoites [27]. The proteins related to function and cytoskeletal function were observed for their relationship with PI3K inhibition [27]. We shortlisted proteins which have an established role in pathways activated during the motility and endocytic processes in amoeba or other related organisms. Further, we chose eight phospho-peptides as possible substrates for EhAGCK1 and EhAGCK2. These phospho-peptides originated from Coactosin (EHI_168340), Unconventional Myosin IB (EHI_110810), RhoGEF-1(EHI_159500), Actophorin(EHI_197480), RhoGEF(EHI_008090), Filamin-A interacting protein (EHI_025370), HEAT repeat domain-containing protein (EHI_050150), and Hypothetical protein (EHI_025430). Sequence details of these peptides are provided in **S2 Table**. The kinase activity of EhAGCK1 and EhAGCK2 for each peptide, measured over a fixed time interval, are depicted in **Fig 4**. The **S5A–S5H Fig** shows kinase activity of EhAGCK1 and EhAGCK2 at various substrate concentration along with kinase-dead mutants as control. The numerical values of data are provided in **S3 Table**. Our result revealed that EhAGCK1 WT specifically phosphorylated RhoGEF (EHI_008090) and Filamin-A-interacting protein (EHI_025370) peptides, while Coactosin (EHI_168340) and Unconventional Myosin IB (EHI_110810) peptides were recognised as substrates by EhAGCK2 WT. Interestingly, peptides derived from the EhHEAT repeat domain-containing protein **(Figs 4 and S5G)** and the Hypothetical protein **(Figs 4 and S5H)** also exhibited notable phosphorylation by EhAGCK1. However, due to the limited information available for these two proteins, the biological significance of this phenomenon remains elusive. These results nonetheless suggest a potentially intriguing pathway orchestrated by AGC kinases in *E*. *histolytica* cell biology. Moreover, these findings provide a foundation for elucidating the downstream effects of EhAGCK1 and EhAGCK2.

### Determination of Michaelis-Menten kinetic parameters of EhAGCK1 and EhAGCK2

**Fig 5:**
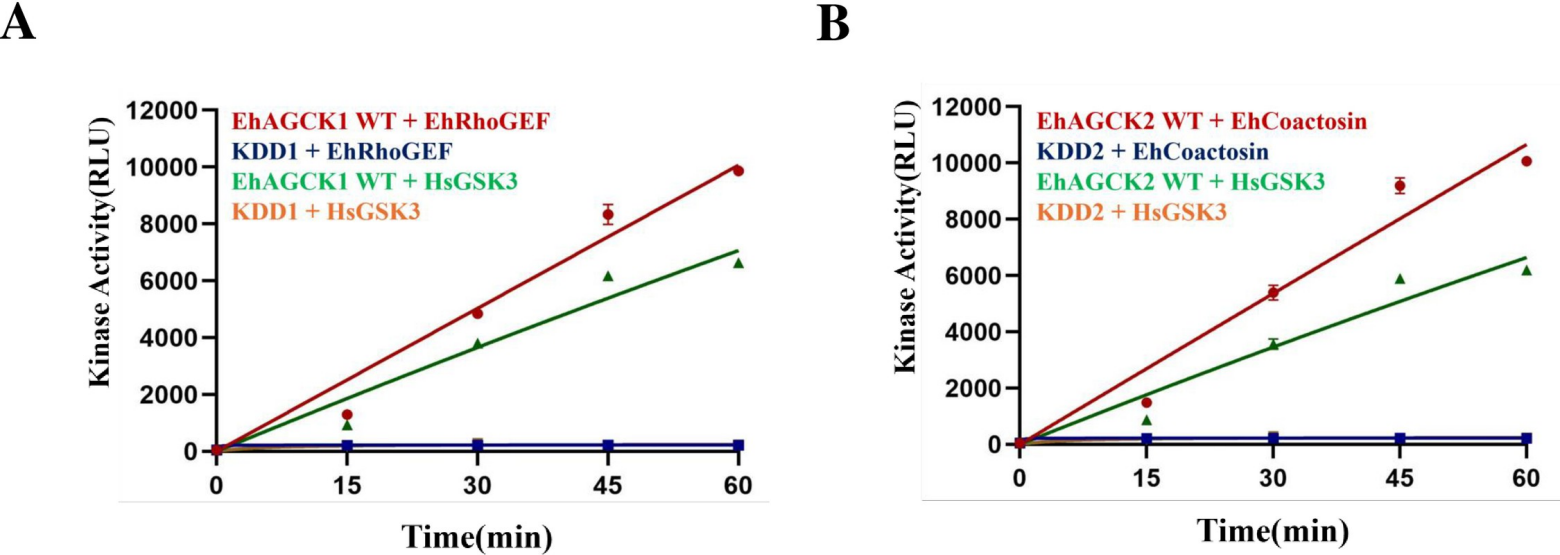
Time point assay to determine the initial rate of kinase reaction. Kinase assay at 20 μM EhAGCK1 (WT and KDD1) and EhAGCK2 (WT and KDD2) was set in the presence of 50 μM peptide substrates and 250 μM ATP at varying time points (0 min–60 min) with 15 min intervals. (A) The graph represents the Michaelis-Menten plot for EhAGCK1 (WT and KDD1) in the presence of EhRhoGEF and HsGSK3 peptide substrates. The X-axis displays the different time points, while the Y-axis shows kinase activity in terms of relative luminescence units (RLU). (B) The graph represents the Michaelis-Menten plot for EhAGCK2 (WT and KDD2) in the presence of EhCoactosin and HsGSK3 peptide substrates. The X-axis displays the different time points, while the Y-axis shows kinase activity in terms of relative luminescence units (RLU). Error bars indicate the mean standard error. Relative luminescence unit (RLU) values are derived from duplicate measurements across three independent experiments (n = 6).

**Fig 6:**
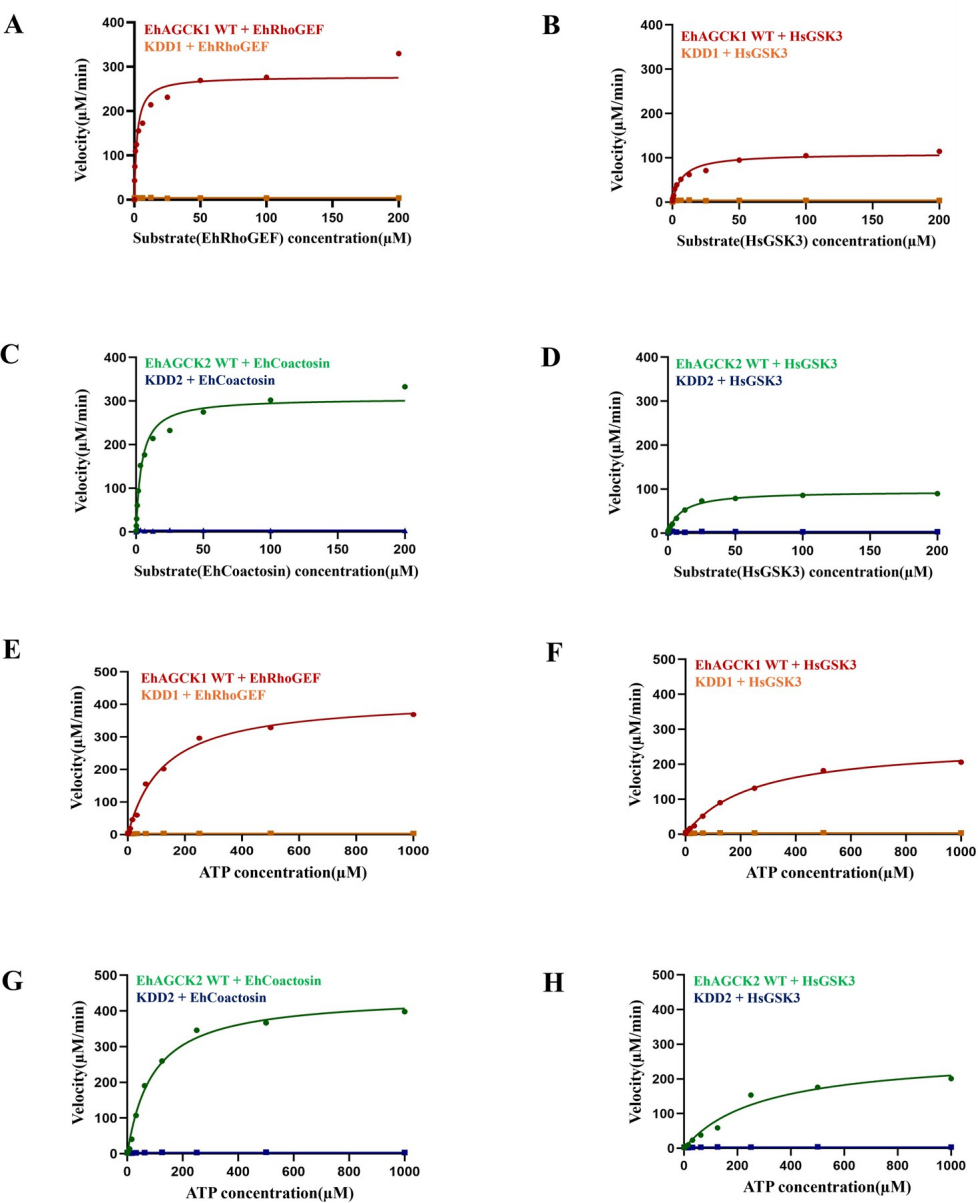
Graphs representing the Michaelis-Menten kinetics of EhAGCK1 and EhAGCK2. Panel A-D represents the ***Km*** and ***Vmax*** for peptide substrates, assays were performed in the presence of 20 μM EhAGCK1 (WT and KDD1) and EhAGCK2 (WT and KDD2) proteins were assayed in the presence of 250 μM ATP and serial dilutions (0 μM to 200 μM) of EhRhoGEF, EhCoactosin, and HsGSK3 peptide substrates. (A) The *Km* of EhRhoGEF for EhAGCK1WT was determined to be 2.07±1.0 μM, and the *Vmax* was 277.8±35.1 μM/min. (B) The *Km* of HsGSK3 for EhAGCK1 WT was determined to be 7.24±1.4 μM, and the *Vmax* was 109±12.06 μM/min. (C) The *Km* of EhCoactosin for EhAGCK2 WT was determined to be 4.10±1.3 μM, and the *Vmax* was 306±24.4 μM/min. (D) The *Km* of HsGSK3 for EhAGCK2 WT was determined to be 9.90±1.6 μM, and the *Vmax* was 95.17±4.4 μM/min. Panel E-H represents the ***Km*** and ***Vmax*** for ATP, assays were assessed in the presence of 50 μM peptide substrates (EhRhoGEF, EhCoactosin, and HsGSK3), serial dilution(0 μM to 1000 μM) of ATP, and 20 μM of the proteins EhAGCK1 (WT, and KDD1) and EhAGCK2 (WT, and KDD2). (E) The *Km* for ATP was determined to be 125.4±16 μM and *Vmax* was 418.9±17.9 μM/min in the presence of EhRhoGEF peptide substrate and EhAGCK1 WT protein. (F) The *Km* for ATP was determined to be 241.7±22.2 μM and the *Vmax* was 260.7±9.4 μM/min in the presence of HsGSK3 peptide substrate and EhAGCK1 WT protein. (G) The *Km* for ATP was determined to be 92.06±16 μM and *Vmax* was 444.4±18.5 μM/min in the presence of EhCoactosin peptide substrate and EhAGCK2 WT protein. (H) The *Km* for ATP was determined to be 291.8±74.4 μM and the *Vmax* was 271.4±30.5 μM/min in the presence of HsGSK3 peptide substrate and EhAGCK2 WT protein. The data were fitted to the nonlinear regression Michaelis-Menten enzyme kinetics model in GraphPad Prism software to obtain the indicated *Km* and *Vmax* values. Relative luminescence unit (RLU) values are converted to μM/min using the method described in the Materials and Methods section. Error bars indicate the mean standard error. Data are derived from duplicate measurements across three independent experiments (n = 6). Note that where error bars are not visible, they are smaller than the symbols.

To determine the Michaelis-Menten constants (*Km*) and maximum reaction rates (*Vmax*) for EhAGCK1 and EhAGCK2, we performed kinase assays with specific peptide substrates (EhRhoGEF, EhCoactosin, HsGSK3) at various time points (0 min-60 min). These data were then plotted against time, resulting in a graph that showed a linear relationship between reaction time and product formation **(Fig 5)**. The *Km* and *Vmax* values for peptide substrates were established using an assay that included a range of concentrations from 0 μM to 200 μM. The *Km* and *Vmax* values for EhAGCK1, using the EhRhoGEF peptide substrate, were determined to be 2.07±1.0 μM and 277.8±35.1 μM/min, respectively. In contrast, when using the HsGSK3 peptide substrate, the *Km* and *Vmax* values were determined to be 7.24±24 μM and 109±12.06 μM/min, respectively **Fig 6A and 6B**. The values of *Km* and *Vmax* for EhAGCK2, using the EhCoactosin peptide substrate, were found to be 4.10±1.3 μM and 306±24.4 μM/min, respectively. The *Km* and *Vmax* values for the HsGSK3 peptide substrate were determined to be 9.90±1.6 μM and 95.17±4.4 μM/min, respectively, as shown in **Fig 6C and 6D**. These results show that amoebic peptide substrates exhibit lower *Km* values compared to human peptide substrates. This is as per our expectation as natural substrates will bind with kinases with more affinity than other similar peptides.

Further, we determined the *Km* and *Vmax* values for ATP as it serves a vital metabolic molecule and substrate crucial for kinase activity, determining ATP *Km* and *Vmax* a key biochemical parameters for all kinases. Consequently, we conducted a kinase assay with various ATP concentrations spanning from 0 μM to 1000 μM. For EhAGCK1, the ATP *Km* and *Vmax* values in the presence of the EhRhoGEF peptide substrate were 125.4±16 μM and 418.9±17.9 μM/min, respectively. Conversely, with the GSK3 peptide substrate, the ATP *Km* and *Vmax* were 241.7±22.2 μM and 260.7±9.4 μM/min, respectively **Fig 6E and 6F**. Similarly, for EhAGCK2, the ATP *Km* and *Vmax* with the EhCoactosin peptide substrate were 92.06±16 μM and 444.4±18.5 μM/min, respectively, while with the GSK3 peptide substrate, they were 291.8±74.4 μM and 271.4±30.5 μM/min, respectively **Fig 6G and 6H**. The values of ATP *Km* for both EhAGCK1 and EhAGCK2 in the presence of native substrates are lower than compared to human GSK3 peptide substrate which is expected for the endogenous substrates. Additionally, we compared previously determined *Km* and *Vmax* values of human Akt proteins in the presence of GSK3 peptide [23] with our kinase protein results, which are presented in **Table 1**. The values are very near to each other hence it is an indication that Akt inhibitors might be modified to inhibit specific amoebic AGC kinase and serve as possible therapeutic agent.

**Table 1:** Table represents the comparison of the *Km* values for the HsGSK3 peptide substrate when subjected to human Akt kinase, as well as the amoebic enzymes EhAGCK1 and EhAGCK2.

S.No.	Protein Name	HsGSK3 peptide substrate *Km*(μM)	ATP *Km*(μM)
1.	Human Akt kinase	3.1±0.6 μM [23]	200±41 μM [23]
2.	EhAGCK1	7.24 ±1.4 μM	241.7 ±22.2 μM
3.	EhAGCK2	9.90 ±1.6 μM	291± 74.4 μM

### Microscale Thermophoresis (MST)

**Fig 7:**
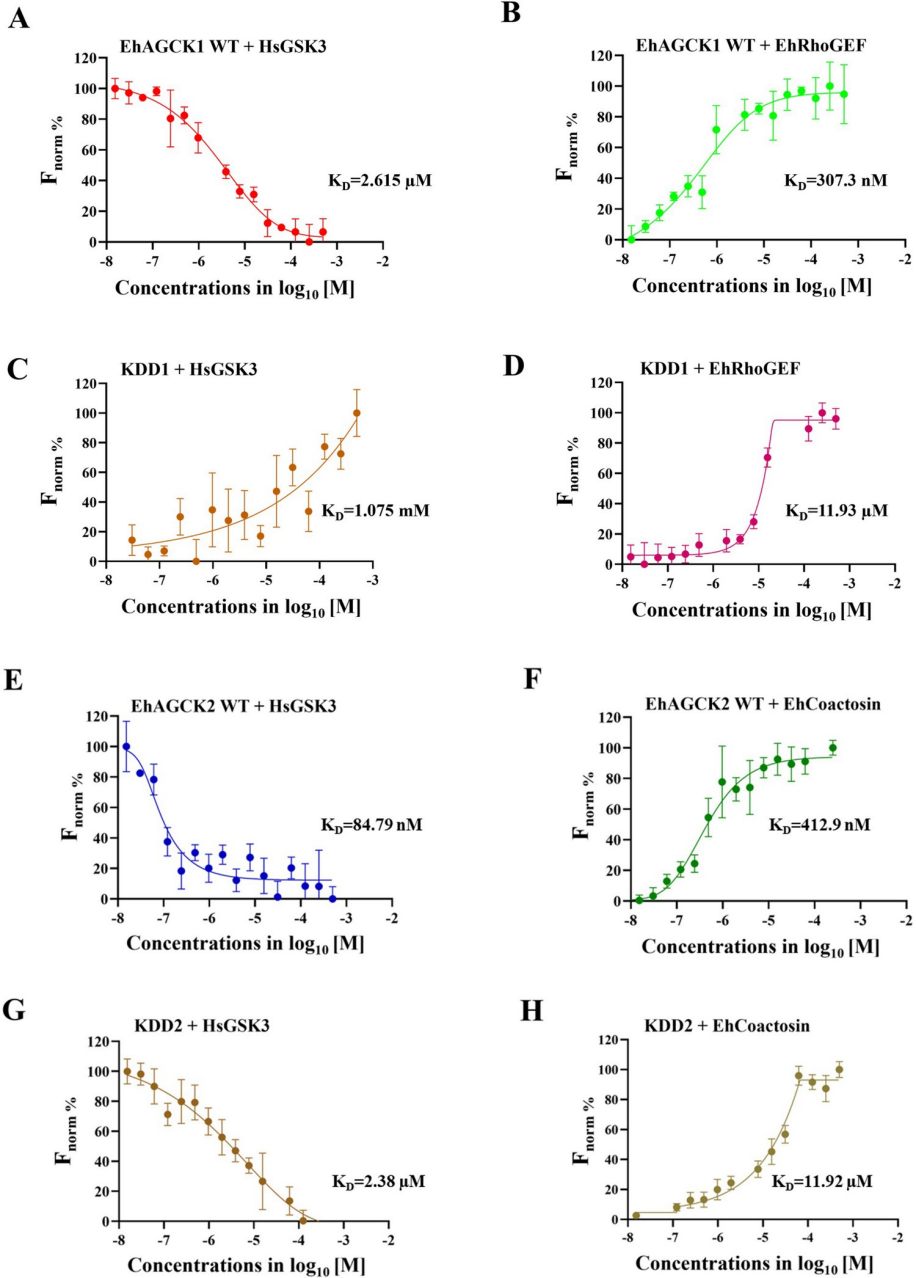
Microscale Thermophoresis (MST) binding studies were conducted in the presence of kinase proteins with peptide substrates. Proteins were labelled using a His-tag RED-tris-NTA 2nd Generation (Nano Temper Technologies) labelling dye. The concentration of proteins was kept constant at 50 nM, while the concentration of the unlabelled peptide substrates varied from higher concentrations to lower concentrations. After a short incubation, the samples were loaded into standard Monolith NT.115 capillaries (Nano Temper Technologies), and the MST measurement was performed using the Monolith NT.115 [NT.115Pico/NT. Label Free] (Nano Temper Technologies). **The graph (A-D)** indicates interaction of EhAGCK1 (WT, and KDD1) protein with peptide substrates including HsGSK3 and EhRhoGEF whereas **graph (E-H)** represents binding of EhAGCK2 (WT, and KDD2) protein with peptide substrates including HsGSK3 and EhCoactosin. The Y-axis represents the ΔF_norm_ fluorescence, and the X-axis represents the concentration of proteins and peptide substrates, respectively. The values presented from duplicate measurements across three independent experiments (n = 6), with error bars representing the mean standard error.

Microscale Thermophoresis (MST) enables the quantification of biomolecular interactions by analysing the movement of biomolecules within a temperature gradient. The applied temperature gradient changes molecular properties such as charge, size, hydration shell, or conformations [28,29]. MST has been widely used to study protein-protein interactions where one protein is fluorescently labelled, and the other is serially diluted. Keeping the concentration of the fluorescently labelled protein constant and creating a concentration gradient for the other protein helps in understanding how the binding affinities between the labelled protein and its binding partner change across the concentration gradient [30,31]. On similar lines, the binding affinities of wild-type EhAGCK1 WT and EhAGCK2 WT proteins, along with their kinase-dead variants (KDD1 and KDD2), were assessed through MST. The results revealed that, for EhAGCK1 WT, the binding constants (K_D_) were 307.3 nM for the EhRhoGEF peptide and 2.615 μM for the HsGSK3 peptide. While EhAGCK2 WT showed affinities of 84.79 nM for the HsGSK3 peptide and 412.9 nM for the EhCoactosin peptide, conversely, both KDD1 and KDD2 exhibited lower affinities compared to their wild-type counterparts. KDD1 bound at 1.075 mM to human GSK3 peptide and 11.93 μM to EhRhoGEF peptide, while KDD2 displayed a 2.38 μM binding affinity to HsGSK3 peptide and 11.92 μM to EhCoactosin. These reduced affinities of the kinase-dead variants are likely attributed to mutations in their ATP binding sites. The dose-response curves for EhAGCK1 WT and KDD1 are shown in **Fig 7A–7D**, whereas the dose-response curves of EhAGCK2 WT and KDD2 are shown in **Fig 7E–7H**, respectively.

### EhPDK1 as upstream activator of EhAGCK1 and EhAGCK2

**Fig 8:**
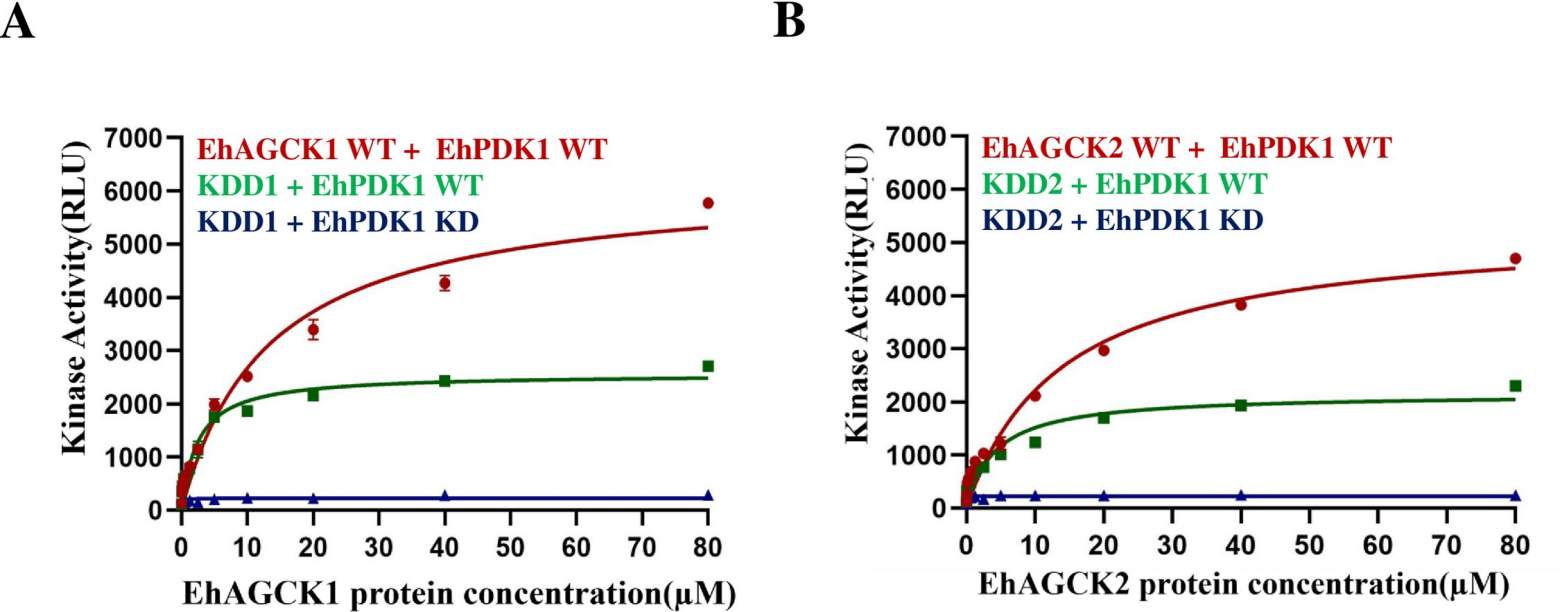
EhPDK1 serves as a common upstream activator for EhAGCK1 and EhAGCK2. Purified recombinant EhAGCK1 (WT and KDD1) and EhAGCK2 (WT and KKD2) proteins were assayed with 50 μM EhPDK1 as described in protocol. (A) The graph shows phosphorylation of EhAGCK1 by EhPDK1 in concentration dependent manner. The red line shows cumulative signal of EhPDK1 phosphorylation of EhAGCK1 along with autophosphorylation by itself. While green line shows substrate phosphorylation activity of EhPDK1 towards KDD1. The X-axis displays the concentration of the protein sample, while the Y-axis expresses kinase activity in terms of relative luminescence units (RLU). (B) The red line on graph shows cumulative signal for EhPDK1 phosphorylation activity towards EhAGCK2 along with autophosphorylation activity of EhAGCK2. The Green line shows phosphorylation signal for EhPDK1 towards kinase deficient KDD2. The X-axis displays the concentration of the protein sample, while the Y-axis expresses kinase activity in terms of relative luminescence units (RLU). The Blue line on graph A and B is control assay in which kinase dead version of both enzymes is used. Error bars indicate the mean standard error. Relative luminescence unit (RLU) values are derived from duplicate measurements across three independent experiments (n = 6).

Our *in-silico* analysis revealed that *E*.*histolytica* PDK1 has a conserved domain similar to human PDK1, which is responsible for ATP binding and phosphorylation. To validate our prediction, we cloned and purified EhPDK1 and performed the kinase assay. The assay was conducted using a fixed concentration of 50 μM EhPDK1 proteins, 250 μM ATP, and serially diluted EhAGCK1 and EhAGCK2 proteins, ranging from 0 μM to 80 μM, in kinase-specific reaction buffers. Our findings demonstrate that full-length EhPDK1 WT phosphorylates both EhAGCK1 WT **(Fig 8A)** and EhAGCK2 WT **(Fig 8B)**. In order to confirm, if EhPDK1 is actually phosphorylating EhAGCK1 and EhAGCK2, additional assays were performed with EhPDK1 WT, KDD1, and KDD2. The KDD1 and KDD2 were phosphate group acceptor without showing any kinase activity of their own and results revealed that EhPDK1 WT is able to phosphorylate KDD1 and KDD2 **(Fig 8A and 8B)**. Hence, we conclude that EhPDK1 serves as a common plausible upstream activator for EhAGCK1 and EhAGCK2. Further, assays with kinase-dead EhPDK1 along with KDD1 and KDD2 serve as negative controls. These findings enhance our understanding of the regulatory mechanisms governing the activity of EhAGCK1 and EhAGCK2, particularly in the context of their interaction with EhPDK1.

### The recombinant EhAGCK1 and EhAGCK2 accepts biotin-ATP as substrate

**Fig 9:**
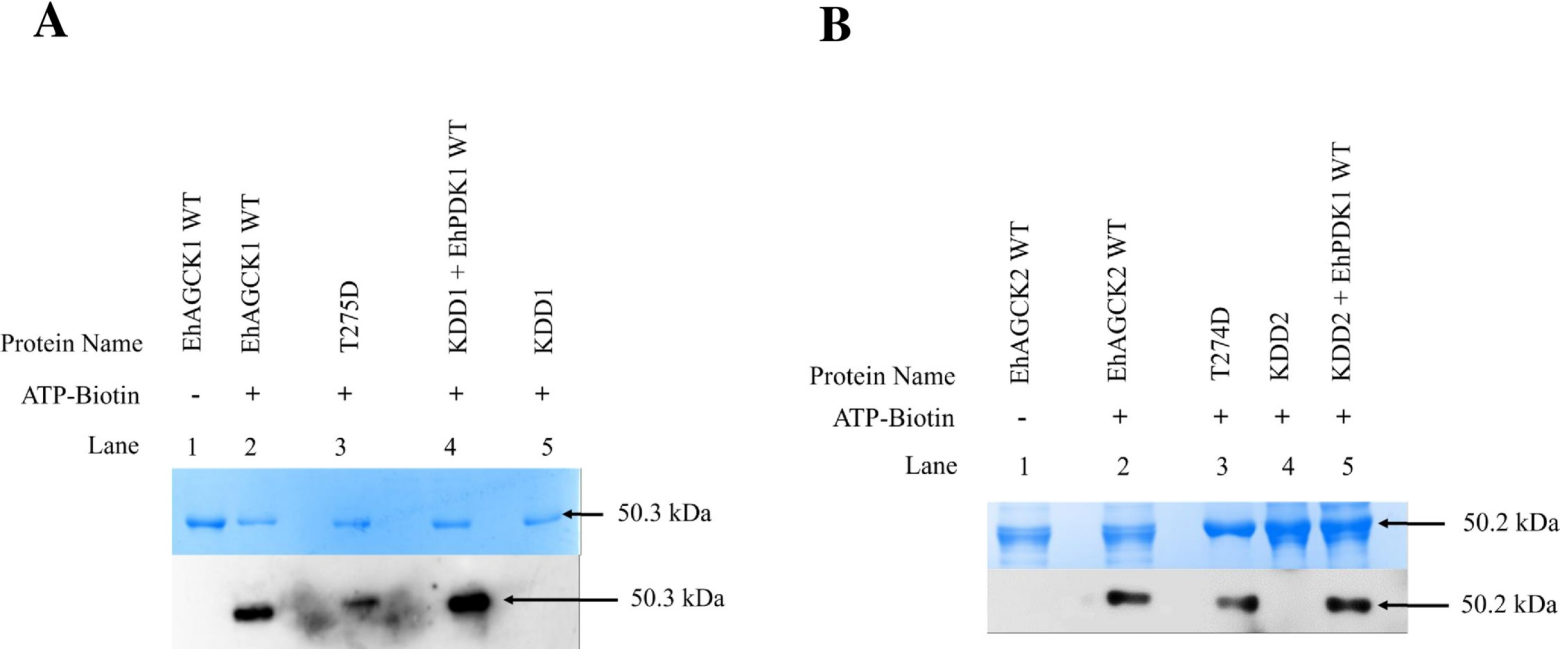
Confirming autophosphorylation by EhAGCK1 and EhAGCK2 and upstream activity of EhPDK1 by Biotin-ATP. **(A) From left to right,** in lane 1, the recombinant purified EhAGCK1WT protein substrate without ATP-biotin was added, while lane 2 contained the same substrate with ATP-biotin. Lane 3 featured constitutively active EhAGCK1 (T275D) alone incubated in ATP-biotin, serving as a positive control. Lane 4 displayed recombinant purified EhPDK1 WT incubated with kinase-dead EhAGCK1 (KDD1), acting as an upstream activator for the EhAGCK1 protein. Finally, lane 5 showed kinase-dead EhAGCK1 (KDD1) alone incubated with ATP-biotin, functioning as a negative control. The reactions were separated by 12% SDS-PAGE and visualized using either Coomassie Blue stain to detect all proteins (top) or western blotted by streptavidin horseradish peroxidase (SA-HRPO) to detect biotinylated proteins (bottom). **(B) From left to right,** lane 1 contains the recombinant purified EhAGCK2 WT protein substrate without ATP-biotin, while lane 2 contains the same substrate with ATP-biotin. In lane 3, constitutively active EhAGCK2 (T274D) alone was incubated in ATP-biotin, serving as a positive control. Conversely, lane 4 features kinase-dead EhAGCK2 (KDD2) alone incubated with ATP-biotin, functioning as a negative control. Finally, in lane 5, recombinant purified EhPDK1 WT was incubated with kinase-dead EhAGCK2 (KDD2), serving as an upstream activator for the EhAGCK2 protein. The reactions were separated by 12% SDS-PAGE and visualized using either Coomassie Blue stain to detect all proteins (top) or streptavidin horseradish peroxidase (SA-HRPO) to detect biotinylated proteins (bottom).

We employed the use of biotin-labelled ATP to discern auto phosphorylation properties of EhAGCK1 and EhAGCK2. The reaction involved the use of ATP, in which gamma phosphate is chemically linked with a biotin tag, which has a high affinity with streptavidin [32]. The kinase-catalyzed ATP-biotinylation was performed with ATP-biotin and full-length recombinant purified proteins. The biotinylated products were separated by SDS-PAGE gel followed with transfer to PVDF membrane and visualized using an SA-HRPO conjugate antibody. The western blot signals revealed that both EhAGCK1 WT and EhAGCK2 WT exhibited autophosphorylation activity **(Fig 9A**; lane 2; and **9B**; lane 2**)**. However, kinase-dead mutants (KDD1 and KDD2) remain inactive **(Fig 9A**; lane 5; and **9B**; lane 4). Whereas constitutively active mutants (T275D and T274D) work as kinase-positive controls and display a biotinylated protein band at the expected molecular weight of 50 kDa **(Fig 9A**; lane 3; and **9B**; lane 3). Along the same line, an experiment was performed with recombinant purified EhPDK1 protein with KDD1 and KDD2 as substrates which lack kinase activity but have intact phosphorylation site for EhPDK1. Biotinylation assay results support our previous finding that EhPDK1 is an upstream activator of EhAGCK1 and EhAGCK2 **(Fig 9A**; lane 4; and **9B**; lane 5). As a control reaction, where ATP was used in place of ATP-biotin, the biotin labelling was not detected **(Fig 9A**; lane 1; and **9B**; lane 1).

## Discussion

*E*. *histolytica* extensively depends on kinases for intracellular signal transduction across multiple pathways. Recent studies have established AGC family kinases, which are subgroups of serine/threonine kinases, as pivotal regulators of critical cellular processes, encompassing signal transduction, cytoskeletal dynamics, and interactions with host cells. However, the precise strategy by which *E*. *histolytica* kill host cells has remained unclear, however, it has been widely speculated that it is a contact-dependent process in which the parasite first destroys the host cells and then engulfs them by phagocytosis. Recently, there has been a paradigm shift in understanding the pathogenicity of this organism with the recognition of a new model of host cell destruction known as trogocytosis [4].

Trogocytosis, a phenomenon observed in *E*. *histolytica*, involves the active consumption of small portions of cellular material from live host cells. This process leads to an increase in calcium levels within the host cells due to decreased membrane stability as a result of multiple bits, ultimately leading to their demise. Interestingly, *E*. *histolytica* exhibits distinct behaviours towards live and pre-killed host cells, with trogocytosis observed exclusively with live cells while pre-killed cells are engulfed via phagocytosis [4,33]. Several molecules, including Gal/NAc lectin, actin, EhC2PK, and PI3K signaling, are implicated in both phagocytosis and trogocytosis. Interference with these proteins has been shown to diminish *E*. *histolytica* pathogenicity and reduce host cell death. Moreover, the type of endocytic process is dictated by membrane receptors, indicating a complex interplay of molecular mechanisms [4,33]. While trogocytosis is also observed in certain immunological cell types and multicellular organisms, it typically does not result in cell killing [34]. In contrast, *E*. *histolytica* trogocytosis is associated with host cell destruction, highlighting its unique contribution to pathogenicity of the parasite [4,33]. This raises questions about the molecular distinctions between similar processes across different species and whether they have evolved to serve species-specific functions.

Utilizing *E*. *histolytica* as a model system, we can investigate these questions step by step, leveraging on its highly endocytic nature. Hypotheses suggest existence of overlapping pathways with unique regulatory molecules, such as EhAGCK1, specific to *E*. *histolytica* trogocytosis while EhAGCK2 is implicated in trogocytosis, phagocytosis, and pinocytosis, supporting this notion [13].

In order to elucidate the molecular mechanisms governed by EhAGCK1 and EhAGCK2 in *E*. *histolytica*, we took biochemical approach combined with computational tools. *In-silico* analysis revealed a notable similarity between amoebic AGC kinases and human Akt kinases [13]. The Akt protein, also known as protein kinase B (PKB), consists of three isoforms: Akt1, Akt2, and Akt3. Activation of Akt kinases is initiated by PI3K signaling in response to extracellular cues, leading to phosphorylation at critical residues, T308 and S473, on Akt1. These phosphorylation events, particularly T308 by PDK1, are crucial for achieving maximal Akt activity [21,35]. Upon activation, Akt phosphorylates various protein targets, including GSK3, thereby modulating diverse cellular processes [36]. Through sequence alignment with PKB/Akt we putatively identified the critical phosphorylation sites as T275 and T430 for EhAGCK1, and T274 for EhAGCK2. Phosphomimetic mutants, named T275D for EhAGCK1 and T274D for EhAGCK2, exhibited increased substrate peptide phosphorylation compared to wildtype proteins. This confirmed that phosphorylation at T275 and T274 in EhAGCK1 and EhAGCK2, respectively, indeed activates the kinase activity of these kinases.

To characterize the kinases biochemically, we optimized conditions for kinase assays using wild-type, constitutively active (T275D and T274D), and kinase-dead (KDD1 and KDD2) mutant proteins. The kinase assay employing a human GSK3 peptide substrate demonstrated that purified recombinant EhAGCK1 and EhAGCK2 are catalytically active and capable of phosphorylating the peptide substrate. Additionally, our results indicated that both kinases exhibit divalent ion specificity for optimal activity. EhAGCK1 displayed maximum kinase activity in the presence of Mn^2+^, whereas EhAGCK2 showed optimal activity with Mg^2+^. Interestingly, EhAGCK1 accepted the human GSK3 peptide as a substrate only in the presence of Mn^2+^, highlighting an observation where an ion dictates substrate recognition by an enzyme in a parasite’s kinase assay. Although it has been previously reported that kinases like EhC2PK and choline and ethanolamine kinases require Mn^2+^ as preferred cofactor, but similar observation has been made in plant kingdom also yet EhAGCK1 represent the peculiar case as another member of the family does not exhibit the similar ion dependence [37–39].

Following the optimization of biochemical conditions for kinase assays with EhAGCK1 and EhAGCK2 proteins, we delved deeper in understanding their involvement in endocytic processes by identifying *in vivo* substrates. As AGC kinases work downstream of PI3K, we thoroughly surveyed the phospho-proteome in response to wortmannin treatment of *E*. *histolytica* trophozoites [27]. We focussed on peptides involved in endocytic processes directly or indirectly in amoeba or related organisms. Our search led us to eight phospho-peptides, which were Coactosin (EHI_168340), Unconventional Myosin IB (EHI_110810), RhoGEF-1 (EHI_159500), Actophorin (EHI_197480), RhoGEF (EHI_008090), Filamin-A interacting protein (EHI_025370), HEAT repeat domain-containing protein (EHI_050150), and Hypothetical protein (EHI_025430). The kinase assays were carried out with selected eight peptides separately to assess their phosphorylation. The results from kinase assay indicated that RhoGEF (EHI_008090) and Filamin-A interacting protein (EHI_025370) peptides were recognized as substrates by EhAGCK1 WT, while Coactosin (EHI_168340) and Unconventional Myosin IB (EHI_110810) peptides were accepted as substrates by EhAGCK2 WT. These findings provide initial insights into potential native downstream substrates for EhAGCK1 and EhAGCK2, emphasizing the necessity for further *in vivo* validation before considering these proteins as definitive substrate candidates.

To further add, our kinase assay data revealed that EhRhoGEF (EHI_008090), identified as a potential substrate for EhAGCK1, was investigated to be an unconventional Rho1 GEF (EhGEF) by our group previously [40]. The EhGEF recruits EhRho1 to the site of phagocytosis in PtdIns(3,4,5)P3 dependent manner and hence leads to spatially restricted activation of EhRho1 [40]. But the regulation of EhGEF is not known and phosphorylation by EhAGCK1 may be crucial for its activation and functioning. This is another interesting aspect which needs more investigation. Furthermore, the phosphorylation of Filamin-A interacting protein (EhFLN) by EhAGCK1 as indicated by kinase assay data may contribute to coupling PI3K signaling to actin binding. Although not much is known about role of EhFLN in amoebic biology but as per literature it is one of crucial protein to stabilize actin structures in cell [41]. As in case of trogocytosis, which is a fast-paced process, the actin remodelling is restricted to small tweezer like appendages directly in contact with live host cell. But mechanism of actin remodelling regulated via binding proteins like EhFLN is required to be investigated in more details.

Bringing focus on EhAGCK2, Unconventional Myosin IB and Coactosin emerged as substrates, both proteins are known to be involved in phagocytosis and implicated in parasite pathogenesis [42,43]. Although it is well known that Unconventional Myosin IB and Coactosin are both phosphorylated *in vivo* as well but their upstream kinase has been revealed in current study only [44].

Coactosin has been known to stabilize actin filaments and its over expression led to decrease in phagocytosis [43]. However, role of phosphorylation in actin remodelling activity is also known in limited details. But it has been reported that increased PI3K activity or increased cellular concentration of PtdIns(3,4,5) leads to increased expression of EhCoactosin (44). Similarly, Unconventional Myosin IB also involved in phagocytosis but regulatory role of phosphorylation in endocytic activity is lacking. However, discovery of these proteins as EhAGCK2 targets suggests its role as an upstream kinase coupling PI3K signaling to actin remodelling during the endocytic pathway [13]. Further experiments are required to elucidate the precise role of phosphorylation in actin dynamics. Nonetheless, our data highlight an important link between the PI3K pathway and various components of actin remodelling in *E*. *histolytica*.

On biochemical front, we determined the *Km* values for ATP and peptide substrates of EhAGCK1 and EhAGCK2. The *Km* values of amoebic peptide substrates, including RhoGEF (2.07±1.0 μM), Filamin-A interacting protein (3.28±1.19 μM), Myosin IB (1.338±0.57 μM), and Coactosin (4.01±1.3 μM), were observed to be lower than those of the human GSK3 peptide for both kinases. Similarly, the *Km* values for ATP while using RhoGEF (125.4±16 μM) and Coactosin (92.06±16 μM) as substrate also followed this trend. The endogenous substrate is expected to show low *Km* values than other acceptors. However, it is noticeable that the ATP *Km* values for EhAGCK1 (241.7±22.2 μM) and EhAGCK2 (291±74.4 μM) observed in our study were marginally higher than the previously reported ATP *Km* value for human Akt kinase (200±41 μM) in the presence of the human GSK3 peptide substrate [23].

Another quantitative parameter to assess the substrate binding affinity to their respective protein kinases, Microscale Thermophoresis (MST) was employed [28]. Our findings indicate that for EhAGCK1, the EhRhoGEF peptide exhibited K_D_ values within the nanomolar range, while the HsGSK3 peptide displayed K_D_ values in the micromolar concentration range. In contrast, for EhAGCK2, both amoebic and human peptide K_D_ values were observed in the micromolar concentration range. This difference may be attributed to the fact that K_D_ is indicator of how tightly the substrate is binding to the enzyme while Km is indicative of both the binding affinity and rate of being processed by enzyme. The specific chemistry of the enzyme’s active site and the interactions between the substrate and the enzyme can influence the relationship between K_D_ and Km. So, it is that EhAGCK1 and EhAGCK2, with their differing amino acid sequences, can exhibit different K_D_ values while maintaining similar Km values. This highlights the complex interplay between binding affinity and catalytic efficiency specific to a subtrate.

As previously mentioned in results, EhAGCK1 and EhAGCK2 sequences contain an activation domain similar to human Akt3 and Akt2 kinases, respectively [13]. This activation is facilitated by EhPDK1 dependent phosphorylation at residues T275 for EhAGCK1 and T274 for EhAGCK2. Homology-based searches indicate the existence of a full-length PDK1 gene (EHI_095940) in *E*. *histolytica* kinome. However, a truncated gene (EHI_006200), likely a pseudogene, was also observed. *In vitro* kinase assays confirmed that recombinant full-length EhPDK1 could phosphorylate kinase-dead mutants of EhAGCK1 and EhAGCK2 as substrates. However, further *in vivo* validation is essential to deepen our understanding of the regulatory mechanisms controlling EhAGCK1 and EhAGCK2 activity, especially concerning their interaction with EhPDK1.

It is known that Akt kinases undergo autophosphorylation in a trans manner at residues T72 and S246 in humans for maximal activity [45]. To understand the autophosphorylation activity of EhAGCK1 and EhAGCK2, a kinase assay was conducted with ATP labelled with biotin at the gamma position. The phosphorylated proteins were probed by western blotting using streptavidin labelled with HRPO enzyme [32]. The western blot analysis confirmed that EhAGCK1 and EhAGCK2 were both able to accept biotin-ATP as substrate and underwent autophosphorylation. Additionally, EhPDK1 was able to transfer a biotin-labelled phosphate group onto EhAGCK1 and EhAGCK2, confirming its role as an upstream activator. However, EhPDK1 did not exhibit any autophosphorylation property toward itself. Hence, AGC kinases exhibit autophosphorylation and also phosphorylated by PDK1 as well *in vitro*, both the phenomenon is very likely to play important role in its regulation.

**Fig 10:**
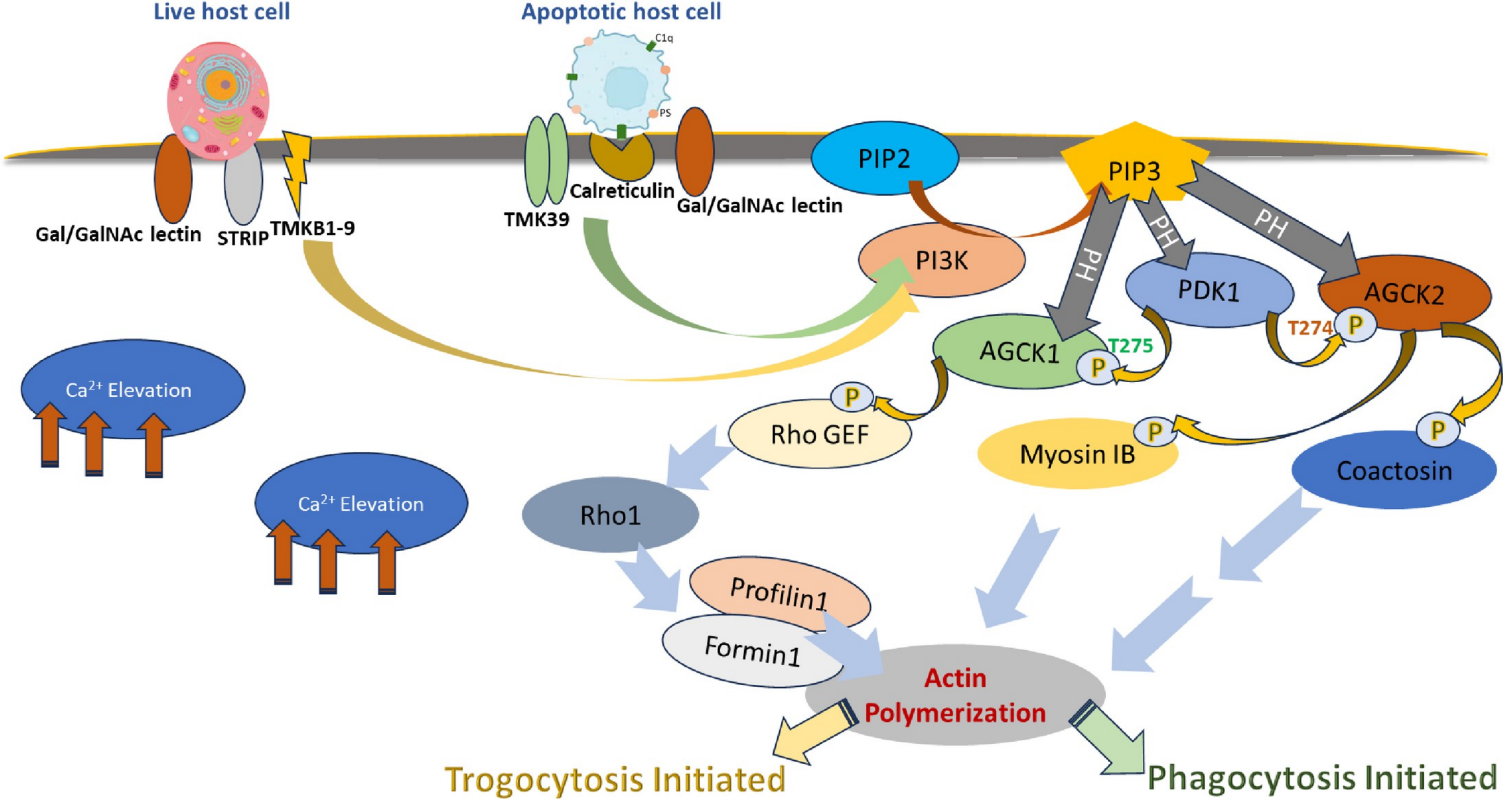
Schematic representation of the pathway depicting role of EhAGCK1 and EhAGCK2 during trogocytosis and phagocytosis. The pathway initiates when *E*. *histolytica* trophozoites attaches to either live or apoptotic host cells, through cell surface receptors like Gal/GalNac lectin [57], which in turn triggers an increase in intracellular calcium levels in trophozoites to through unknown mechanism. This calcium influx regulates downstream signaling molecules such as Phosphoinositide 3-Kinase (PI3K), which converts PtdIns(3,4)P2 to PtdIns(3,4,5)P3. Generation of PtdIns(3,4,5)P3 at the site of phagocytosis or trogocytosis leads to recruitment of EhPDK1, EhAGCK1 and EhAGCK2 proteins at the plasma membrane. The molecular crowding facilitates EhPDK1 to phosphorylate EhAGCK1 at T275 and EhAGCK2 at T274 in the "activation loop," resulting in their activation. Subsequently, activated kinases phosphorylate specific substrates: EhAGCK1 phosphorylates EhRhoGEF (EH_008090), leading to nucleotide exchange on EhRho1, while EhAGCK2 targets EhCoactosin (EHI_168340) and EhMyosin IB (EHI_110810) as downstream substrates. These phosphorylated substrates modulate actin dynamics: EhRhoGEF influences actin dynamics through EhRho1 in EhFormin1 and EhProfilin1 dependent manner, EhCoactosin stabilizes actin filaments, and EhMyosin IB interacts with the Arp2/3 complex [40,43,58]. Downstream of EhAGCK1, trogocytosis is induced, while the actions of EhAGCK2 regulate various endocytic processes, including phagocytosis, trogocytosis, and pinocytosis.

In summary, our combined biochemical and biophysical approaches have provided preliminary insights into connecting the PI3K pathway to actin remodelling proteins via EhAGCK1 and EhAGCK2. Based on our *in vitro* kinase assay data, we have formulated a model illustrating the convergence of phagocytosis and trogocytosis on actin dynamics **(Fig 10)**. Further, our study has generated new leads for investigating pathways regulating different endocytic processes. Furthermore, exploring these underlying mechanisms holds promise in identifying novel therapeutic targets for the development of innovative treatments. Thus, elucidating these intricate pathways not only contributes to advancing our understanding of *E*. *histolytica* biology but also has significant implications for therapeutic intervention strategies in other diseases as well. These findings hold promise for the development of anti-parasitic agents to combat amebiasis and related parasitic diseases in times when increased drug tolerance against metronidazole has already been reported [46]. Our study has also relevance in terms of understanding the evolutionary trends of endocytic processes involved in antigen presentation, cellular autophagy during development which are phenotypically very similar to trogocytosis but serve different purpose [47]. It will help in recognizing the differences or similarities in phenotypically same processes to address care for autoimmune diseases and parasite infection like *Trichomonas vaginalis* which escape immune system by using trogocytosis [48].

## Materials and methods

### Structural modeling of EhAGCK1, EhAGCK2 and EhPDK1

The three-dimensional structures of EhAGCK1, EhAGCK2 and EhPDK1 were modeled using AlphaFold3 [49]. For further refinement, the structures were subjected to 200ns molecular dynamics simulations using the GROMACS software suite as reported previously [50]. The structures of EhAGCK1 and EhAGCK2 were aligned to human Akt1 structure (PDB ID:3OCB and 6HHG) using TM-Align [51,52]. The structural comparison of EhPDK1 with human PDK1(PDB ID:3HRF) was done similarly [53] Visual analysis was done using PyMOL [54].

### Cloning of various constructs used

For the construction of bacterial protein expression plasmid, the EhAGCK1(EHI_188930), EhAGCK2(EHI_053040) and EhPDK1(EHI_095940) protein-coding regions were amplified by PCR from cDNA using specific oligonucleotide containing appropriate restriction site. A PCR amplified DNA fragment of EhAGCK1 and EhAGCK2 were cloned in His-tag pCold protein expression vector whereas EhPDK1 was cloned in GST-tag pGEX-6P-1 protein expression vector. Kinase-dead mutants and constitutively active mutants of EhAGCK1 and EhAGCK2 were generated by mutating the conserved lysine and aspartate residues critical for protein kinase activity by site-directed mutagenesis. The conserved residues lysine(K147) and aspartate(D241) were mutated to alanine (K147A and D241A) in the case of EhAGCK1. In EhAGCK2 conserved residues lysine(K147) and aspartate(D242) were mutated to alanine (K147A and D242A). Kinase-dead mutant of EhPDK1 was generated by mutating lysine(K37) and aspartate(D131) to alanine (K37A and D131A). In EhAGCK1 constitutively active mutant was generated by mutating the threonine(T275) to aspartate(T275D), in the case of EhAGCK2 threonine (T274) was mutated to aspartate(T274D). All the constructs were sequenced and confirmed before being used.

### Protein expression and purification

For protein expression, *Escherichia coli* strain of BL21(DE3) and C41(DE3) was transformed using the pCold-His-EhAGCK1 and EhAGCK2 plasmid and pGEX6P-1 GST-EhPDK1. The transformants were selected using LB agar containing 100 μg/mL of ampicillin. Isolated colonies were cultured in LB medium with 100 μg/mL of ampicillin and incubated at 37°C with shaking. A 1-L culture was inoculated and incubated in a shaker at 37°C until reaching an optical density at 600 nm (OD_600_) of 0.7. The culture was flash-cooled in an ice water bath for 30 min. Induction of protein expression was made by adding 0.5 mM isopropyl-β-d-thiogalactopyranoside (IPTG) (Sigma) to the medium followed by incubation at 15°C with shaking. The best yield of protein was obtained when cultures were harvested 18 h after IPTG induction. Cells were collected and dissolved in lysis buffer; 50mM Tris–HCl, pH 8, 150mM NaCl, and 1mM beta-mercaptoethanol (β-ME), 5% glycerol, 100 ug/ml lysozyme, 1mM phenylmethylsulfonyl fluoride (PMSF), 0.1% tritonX-100. The protein expression was confirmed by loading the soluble and insoluble fractions in SDS-PAGE, followed by Coomassie blue staining and anti-His immunoblot analysis. His-EhAGCK1 and EhAGCK2 were purified by binding with Ni^2+^-NTA His-binding slurry (Qiagen, Germany) and eluting with imidazole at 100mM concentration whereas GST-EhPDK1 was purified with GST binding slurry(Qiagen, Germany). Purified proteins were stored at −80°C with 20% glycerol in small aliquots until use.

### ADP-Glo kinase assay

The phosphorylation activity of kinases was measured using the ADP-Glo kinase assay kit according to the manufacturer’s protocol with few modifications (Promega, Madison, WI, Catalogue number V6930). Assays were performed in a Corning 384-well low-volume white round bottom polystyrene NBS microplate with a total volume of 32 μl in the ratio of 1:1:2, corresponding to the kinase reaction, ADP-Glo reagent, and detection reagent, respectively. The kinase reaction was performed in an 8 μl volume containing 4 μl of purified protein kinases, 2 μl of peptide substrate, and 2 μl of ATP (Promega, Madison, WI). The working concentration of all the kinase reaction components was prepared in the standard kinase assay buffer (40 mM Tris-Cl pH 7.5, 20 mM MgCl_2_, 2 mM DTT). Along with other controls, only buffer-negative controls without protein kinase, substrate, and ATP were included in the kinase assay. Kinase assay reactions in each well were started immediately by adding ATP. The reactions were allowed to continue for 1 h at 30°C in the incubator chamber without shaking. The kinase assay was quenched by adding 8 μl of ADP-Glo reagent in each well before adding ADP-Glo reagent. The reaction plate was incubated for 5 min at room temperature. Termination reactions were allowed to proceed for 40 min at room temperature. ADP-Glo reagent, in addition to quenching the reaction, scavenges the unconsumed ATP from the reaction. In the final step of the ADP-Glo kinase assay, 16 μl of kinase detection reagent was added to each well and incubated for 40 min to simultaneously convert ADP to ATP and allow the newly synthesised ATP to be measured using a luciferase/luciferin reaction. The luminescent signal generated was measured using a SpectraMax i3x Multi-Mode Microplate Reader (Molecular Devices), and it was proportional to the ADP concentration produced and correlated with the kinase activity.

### Microscale Thermophoresis (MST)

Microscale Thermophoresis (MST) was performed in the presence of full-length and kinase-dead EhAGCK1 and EhAGCK2 proteins, along with HsGSK3, EhRhoGEF, and EhCoactosin peptide substrates. Proteins were labelled using a His-tag labelling kit, RED-tris-NTA 2nd Generation (Nano Temper Technologies, San Francisco, CA, USA). A brief labelling reaction was performed in a buffer containing (50 mM Tris-Cl pH 8, 150 mM NaCl, 1 mM β-ME and 5% glycerol) with a protein concentration of 20 nM and a molar dye: protein ratio of approximately 3:1. The mixture was kept at room temperature for 30 min, followed by centrifugation at 15000 g for 10 min at 4°C. A series of 16 1:1 dilutions were prepared using the same buffer, resulting in peptide concentrations ranging from 500 μM to 153 nM. Following a 10 min incubation, the samples were loaded into standard monolith NT.115 capillaries from Nano Temper Technologies. MST measurements were conducted using a Monolith NT.115 instrument at an ambient temperature of 25°C, with instrument parameters set to 50% LED and medium MST power. Data from three independently pipetted measurements were analysed using MO. Affinity Analysis software version 2.1.3 from Nano Temper Technologies focuses on the signal obtained during an MST on time of 5 s.

### ATP biotinylation kinase assay

ATP biotinylation assays were performed in the presence of 4 μM of EhAGCK1 (WT, T275D, and KDD1) and EhAGCK2 (WT, T274D, and KDD2) along with 2 μM of wild-type EhPDK1 in the presence of activation buffer 40 mM Tris-Cl pH 7.8, 10 mM MgCl_2_, and 2 mM DTT and incubated at 30°C for 1 h. The reaction was triggered by adding 20 μM biotin-labelled ATP (Catalogue NU-970-BIO, Jena Bioscience). 20 μl of the reaction was collected at the indicated time point and quenched by the addition of 4x SDS-loading dye. After gel electrophoresis, phosphorylated proteins were visualized by immunoblotting using HRPO-conjugated streptavidin (Invitrogen).

### Conversion of rates from RLU (relative luminescence units) to μM/min

Standard curves were constructed by plotting the saturating relative light units (RLU) for each sample against the concentration of the peptide substrate or ATP in the reaction, following the previously reported method [55]. The slope of the standard curve was determined and used to convert the reaction rate velocities. Velocities were expressed in μM/min by calculating the rate of reaction (DY/DX) for each reaction, where DY represented the difference between the luminescence measurements at the beginning and end of the reaction, and DX denoted the duration of the reaction (total time of reaction: 60 min). The converted velocities were plotted against the substrate concentration, and the data were fitted to the Michaelis-Menten equation to determine *Km* and *Vmax*.

### Western blotting

ATP biotinylated protein samples were separated by SDS-PAGE and then electro-transferred to PVDF membrane (Pall life sciences). The membrane was blocked overnight by 5% BSA (G-Biosciences) in TBS-T (TBS with 0.1% Tween 20) and stained with Ponceau S. This blocking process was done overnight at 4°C. The membrane was then incubated with 1:1000 dilution of HRPO-conjugated streptavidin (Invitrogen) at room temperature. Following antibody incubation, the blots were carefully cleaned with TBS-T. Band detection was carried out with a Millipore ECL kit.

### Statistical analysis

The kinase assay data presented here are the mean ± standard error (SE) of n≥3 of three independent experiments. The *Km* and *Vmax* were determined from a direct, nonlinear fit of [V] versus [S] plots using the Michaelis-Menten equation: V=Vmax[S]/Km+[S] using the GraphPad Prism 9.0 software package (GraphPad Software Inc., CA, USA). Additionally, the Microscale Thermophoresis (MST) experiments were conducted using the Monolith NT.11.5 instrument, with triplicate runs. Statistical comparisons were made using Student’s t-test. Differences in mean values were considered significant at *p-value≤0.05, **p-value≤0.01, ***p-value≤0.001, ****p-value≤0.0001.

## Supporting information

S1 Fig Sequence alignment of EhAGCK1 and EhAGCK2 with human Akt1. The alignment reveals that the domains and motifs essential for ATP binding and the activation loop for Ser/Thr protein kinase phosphorylation region are conserved between human Akt1 and EhAGCK1 and EhAGCK2.
(TIF)

S2 Fig (A) Sequence similarities between EhPDK1(EHI_095940) and EhPDK2(EHI_006200). Sequence alignment of EhPDK1 (EHI_095940) and EhPDK2 (EHI_006200) reveals high similarity at the N-terminal end and conserved PH domain at C-terminal end. **(B) Schematic representation of EhPDK1 (EHI_095940) and EhPDK2 (EHI_006200).** In EhPDK2 (EHI_006200), the kinase domain is lacking essential activation motif. The figure shows amino acid length spanning the domains in the sequence. **(C) Sequence alignment of human PDK1 (CAG38755.1) and EhPDK1 (EH_095940).** The alignment shows domains and motifs essential for ATP binding are conserved between human PDK1 (CAG38755.1) and EhPDK1 (EH_095940). Conserved residues are marked with asterisks, and similar residues are marked with dots.
(TIF)

S3 Fig Expression and purification of recombinant EhAGCK1, EhAGCK2 and EhPDK1 protein. SDS PAGE gel showing (A) Recombinant full length EhAGCK1 His-tagged protein. The lanes, from left to right, represent pre-stained protein marker (lane 1) and full-length His-tagged EhAGCK1 protein (lane 2). (B) Full length His-tagged EhAGCK2 recombinant protein. The lanes, from left to right, represent pre-stained protein marker (lane 1) and full-length His-tagged EhAGCK2 protein (lane 2). (C) Full length GST-tagged EhPDK1 recombinant protein. The lanes, from left to right, represent pre-stained protein marker (lane 1) and full-length GST-tagged EhPDK1 protein (lane 2).
(TIF)

S4 Fig Activation of kinase activity of EhAGCK1 and EhAGCK2 in the presence of MgCl_2_ and MnCl_2_. (A) Graph shows lack of kinase activity of EhAGCK1 with HsGSK3 peptide substrate in the presence of MgCl_2_ buffer. (B) The graph shows kinase activity of EhAGCK2 towards HsGSK3 peptide in MgCl_2_ Buffer. (C) The graph showing significant increase in kinase activity of EhAGCK1 towards HsGSK3 peptide in presence of Mn^2+^ ion, while kinase activity of EhAGCK2 remains unaffected as seen in graph (D). On the X axis, the concentration of the protein sample is displayed and on the Y axis, kinase activity in terms of the relative luminescence unit (RLU) is expressed. The data were fitted to the nonlinear regression Michaelis-Menten enzyme kinetics model in GraphPad Prism software to obtain the indicated *Km* and *Vmax* values. Error bars indicate the mean standard error. Relative luminescence unit (RLU) values are derived from duplicate measurements across three independent experiments (n = 6).
(TIF)

S5 Fig Screening of *E*. *histolytica* peptides for phosphorylation. The graph (A-H) shows the kinase activity of EhAGCK1 and EhAGCK2 with selected *E*.*histolytica* peptide substrates as labelled below the graphs. The data were fitted to the nonlinear regression Michaelis-Menten enzyme kinetics model in GraphPad Prism software to obtain the indicated *Km* and *Vmax* values. Error bars indicate the mean standard error. Relative luminescence unit (RLU) values are derived from duplicate measurements across three independent experiments (n = 6).
(TIF)

S1 Table A comparative analysis of kinase activity fold change in the presence of MgCl_2_ and MnCl_2_ buffers. (DOCX)

S2 Table List of commercially synthesized peptide substrates used in this study. (DOCX)

S3 Table Table illustrating the Michaelis-Menten constant (*Km*) of various amoebic peptide substrates for EhAGCK1 and EhAGCK2. (DOCX)
