# Supplementary figures and images for: AGC family kinase of Entamoeba histolytica: Decoding the members biochemically
Source: PLoS Pathog. 2024 Nov 19;20(11):e1012729. doi: 10.1371/journal.ppat.1012729 (PMC11642994; doi:10.1371/journal.ppat.1012729)

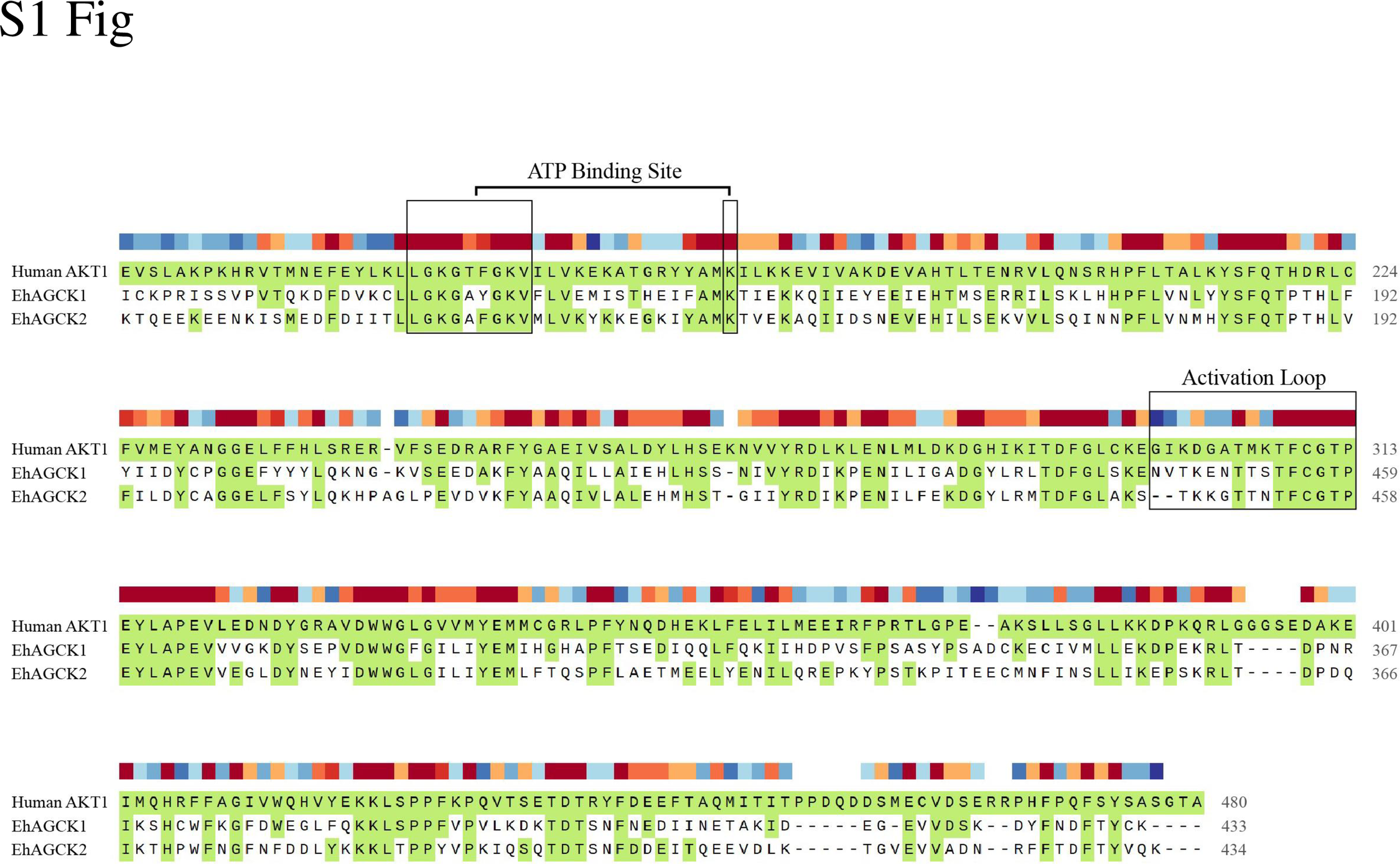

Supplement: S1 Fig — The alignment reveals that the domains and motifs essential for ATP binding and the activation loop for Ser/Thr protein kinase phosphorylation region are conserved between human Akt1 and EhAGCK1 and EhAGCK2. (TIF) [file ppat.1012729.s001.tif]

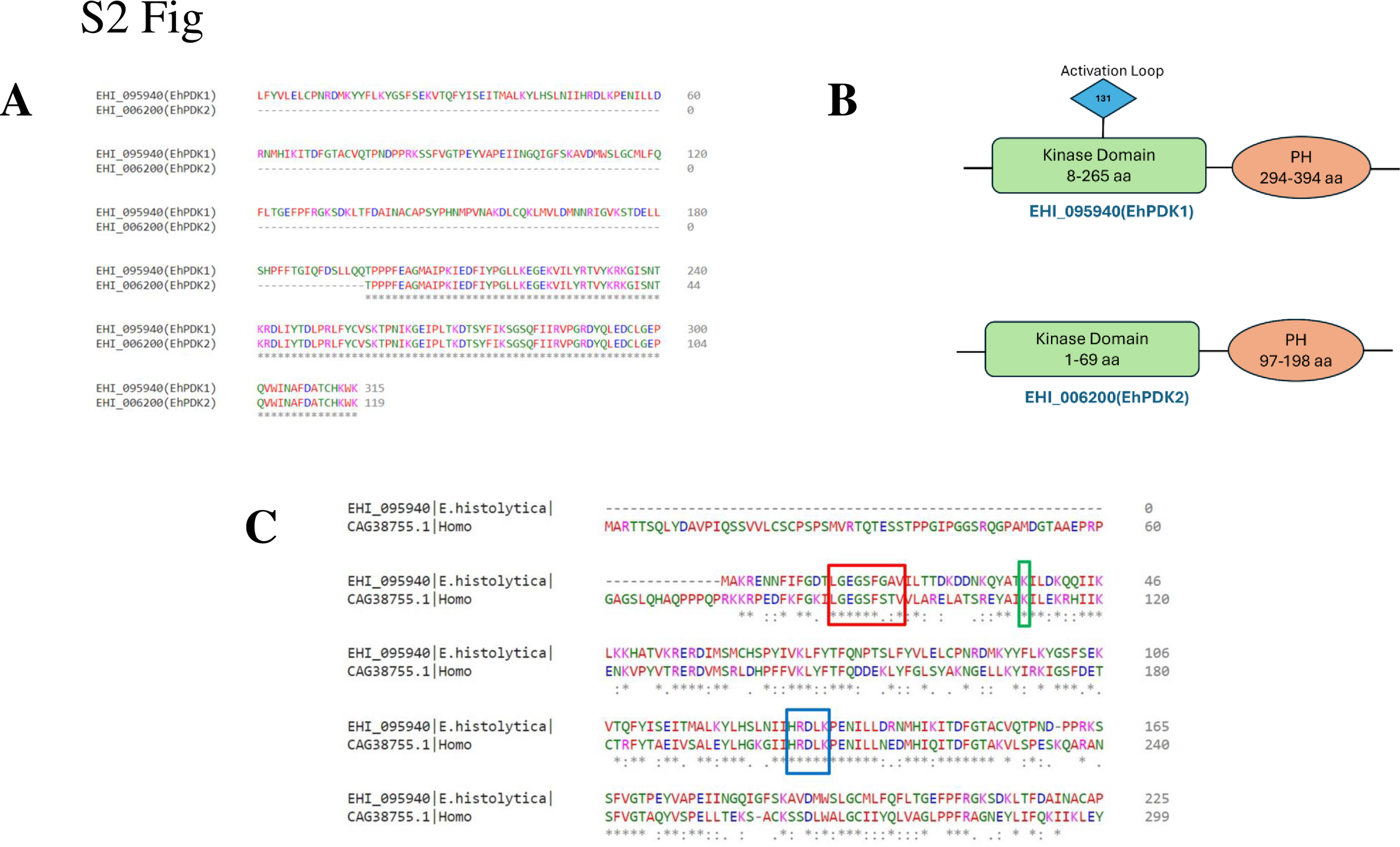

Supplement: S2 Fig — Sequence alignment of EhPDK1 (EHI_095940) and EhPDK2 (EHI_006200) reveals high similarity at the N-terminal end and conserved PH domain at C-terminal end. (B) Schematic representation of EhPDK1 (EHI_095940) and EhPDK2 (EHI_006200). In EhPDK2 (EHI_006200), the kinase domain is lacking essential activation motif. The figure shows amino acid length spanning the domains in the sequence. (C) Sequence alignment of human PDK1 (CAG38755.1) and EhPDK1 (EH_095940). The alignment shows domains and motifs essential for ATP binding are conserved between human PDK1 (CAG38755.1) and EhPDK1 (EH_095940). Conserved residues are marked with asterisks, and similar residues are marked with dots. (TIF) [file ppat.1012729.s002.tif]

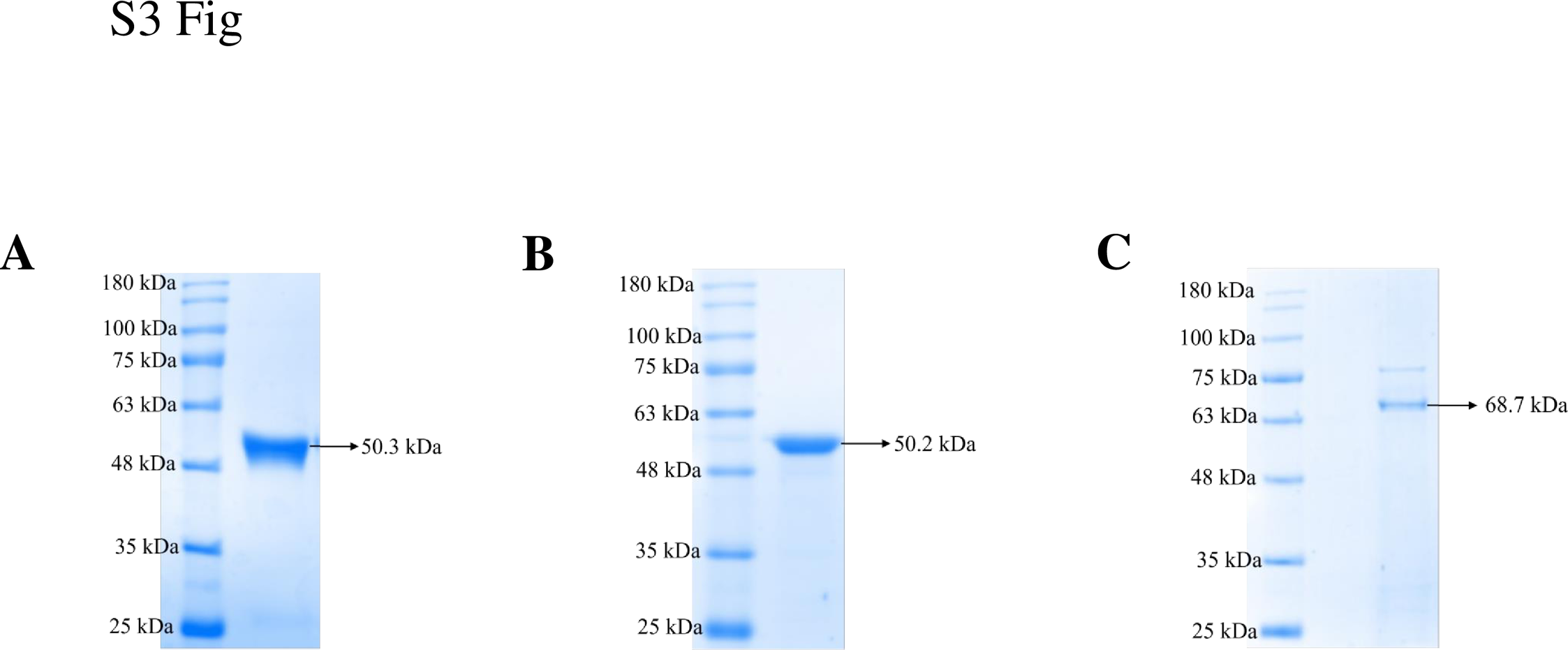

Supplement: S3 Fig — SDS PAGE gel showing (A) Recombinant full length EhAGCK1 His-tagged protein. The lanes, from left to right, represent pre-stained protein marker (lane 1) and full-length His-tagged EhAGCK1 protein (lane 2). (B) Full length His-tagged EhAGCK2 recombinant protein. The lanes, from left to right, represent pre-stained protein marker (lane 1) and full-length His-tagged EhAGCK2 protein (lane 2). (C) Full length GST-tagged EhPDK1 recombinant protein. The lanes, from left to right, represent pre-stained protein marker (lane 1) and full-length GST-tagged EhPDK1 protein (lane 2). (TIF) [file ppat.1012729.s003.tif]

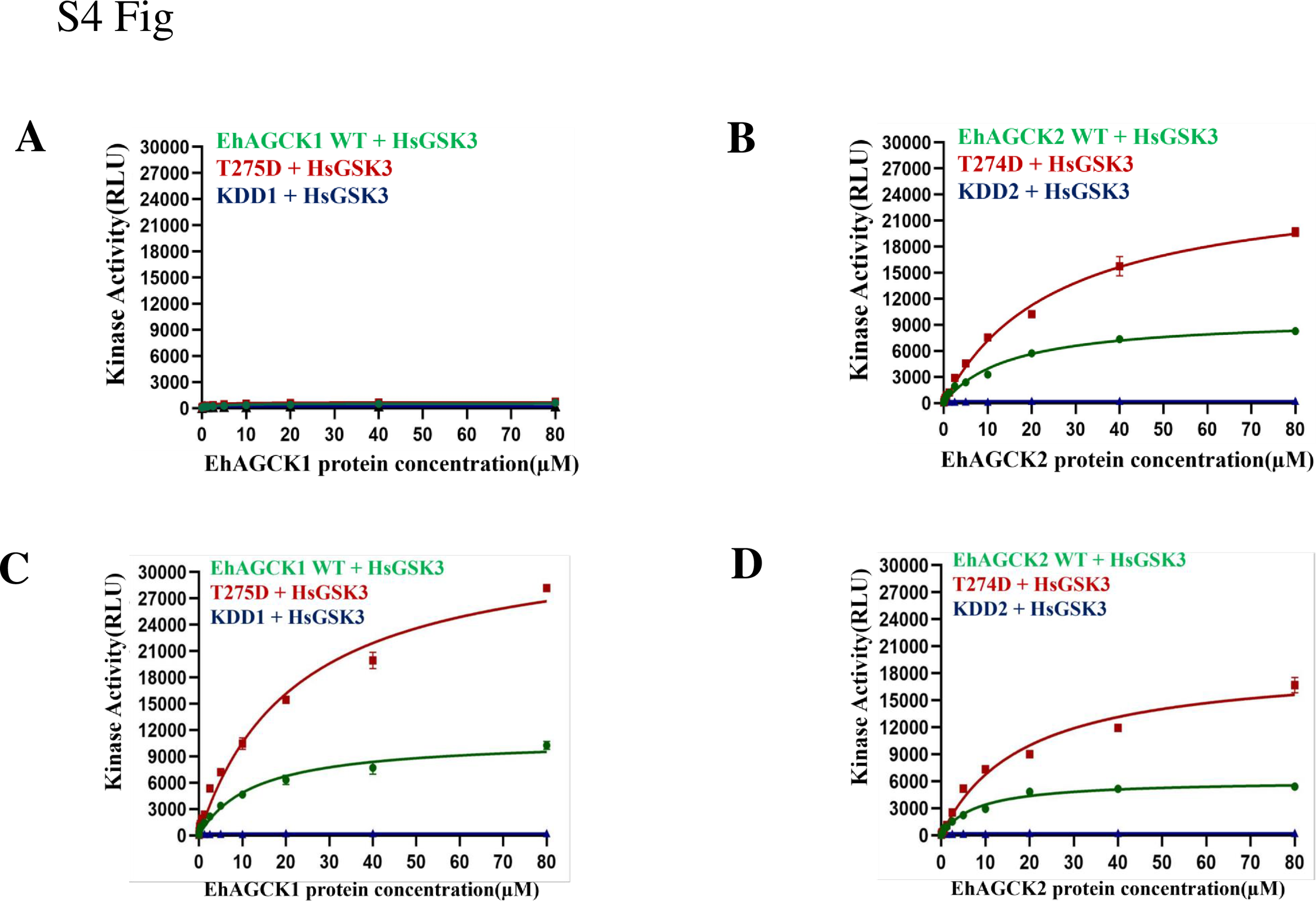

Supplement: S4 Fig — (A) Graph shows lack of kinase activity of EhAGCK1 with HsGSK3 peptide substrate in the presence of MgCl2 buffer. (B) The graph shows kinase activity of EhAGCK2 towards HsGSK3 peptide in MgCl2 Buffer. (C) The graph showing significant increase in kinase activity of EhAGCK1 towards HsGSK3 peptide in presence of Mn2+ ion, while kinase activity of EhAGCK2 remains unaffected as seen in graph (D). On the X axis, the concentration of the protein sample is displayed and on the Y axis, kinase activity in terms of the relative luminescence unit (RLU) is expressed. The data were fitted to the nonlinear regression Michaelis-Menten enzyme kinetics model in GraphPad Prism software to obtain the indicated Km and Vmax values. Error bars indicate the mean standard error. Relative luminescence unit (RLU) values are derived from duplicate measurements across three independent experiments (n = 6). (TIF) [file ppat.1012729.s004.tif]

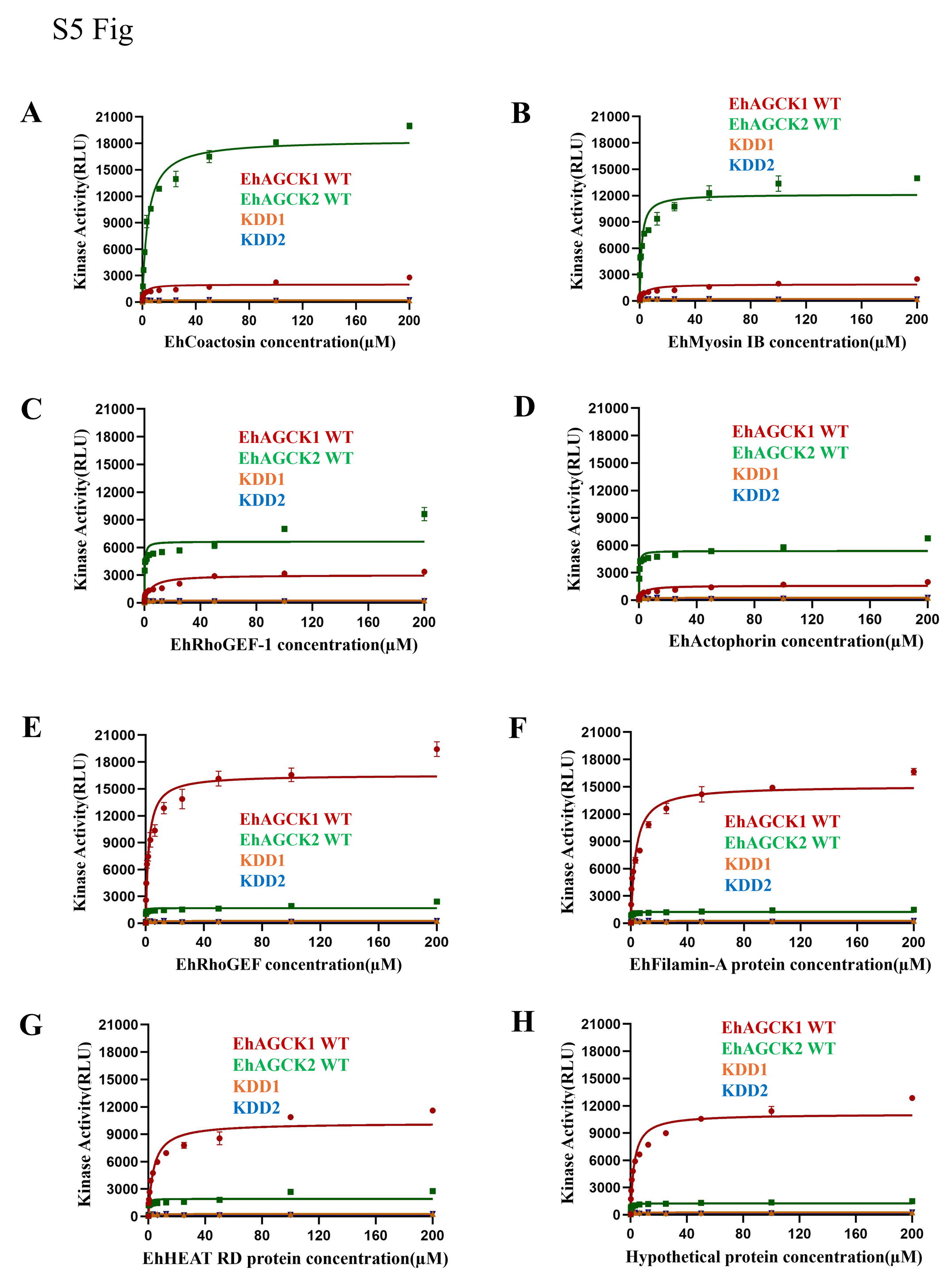

Supplement: S5 Fig — The graph (A-H) shows the kinase activity of EhAGCK1 and EhAGCK2 with selected E.histolytica peptide substrates as labelled below the graphs. The data were fitted to the nonlinear regression Michaelis-Menten enzyme kinetics model in GraphPad Prism software to obtain the indicated Km and Vmax values. Error bars indicate the mean standard error. Relative luminescence unit (RLU) values are derived from duplicate measurements across three independent experiments (n = 6). (TIF) [file ppat.1012729.s005.tif]
