# Supplementary material for: AGC family kinase of Entamoeba histolytica: Decoding the members biochemically
Source: PLoS Pathog. 2024 Nov 19;20(11):e1012729. doi: 10.1371/journal.ppat.1012729 (PMC11642994; doi:10.1371/journal.ppat.1012729)
Supplement: S1 Table — (DOCX) [file ppat.1012729.s006.docx]

**S1 Table**

| Names of kinase variants | Fold change (MgCl_2_: MnCl_2_) |
| --- | --- |
| EhAGCK1 WT | 15.67 |
| T275D | 36.35 |
| EhAGCK2 WT | 0.63 |
| T275D | 0.82 |
