# Supplementary material for: AGC family kinase of Entamoeba histolytica: Decoding the members biochemically
Source: PLoS Pathog. 2024 Nov 19;20(11):e1012729. doi: 10.1371/journal.ppat.1012729 (PMC11642994; doi:10.1371/journal.ppat.1012729)
Supplement: S2 Table — (DOCX) [file ppat.1012729.s007.docx]

**S2 Table**

| Names of peptide substrates | Peptide Sequence | Position of peptide sequence in protein sequence | Organism | Accession Number | Manufacture’s detail |
| --- | --- | --- | --- | --- | --- |
| GSK3 | GRPRTTSFAESCK |  | Human |  | Pepmic Peptide LLP(China) |
| Coactosin | KAGGADYSFNTTSN | 135-148 | *E. histolytica* | EHI_168340 | Priveel Peptide LLP(India) |
| Unconventional myosin IB | RRESVDYTNNK | 754-764 | *E. histolytica* | EHI_110810 | Priveel Peptide LLP(India) |
| RhoGEF-1 | SALDNGTYSPLLSPTR | 236-251 | *E. histolytica* | EHI_159500 | Priveel Peptide LLP(India) |
| Actophorine | MAGIQLADEVTSVYNDF  KLSHKYRYIV | 1-27 | *E. histolytica* | EHI_197480 | Priveel Peptide LLP(India) |
| RhoGEF protein | SSPSSVPAAQSTTPVNVPK | 114-132 | *E. histolytica* | EHI_008090 | Priveel Peptide LLP(India) |
| Filamin-A interacting protein | ELDSDEEQKELER | 407-419 | *E. histolytica* | EHI_025370 | Priveel Peptide LLP(India) |
| HEAT repeat domain-containing protein | KTITPVVEDPCER | 661-673 | *E. histolytica* | EHI_050150 | Priveel Peptide LLP(India) |
| Hypothetical protein | KSPTIDEIK | 133-141 | *E. histolytica* | EHI_025430 | Priveel Peptide LLP(India) |
