# Supplementary material for: AGC family kinase of Entamoeba histolytica: Decoding the members biochemically
Source: PLoS Pathog. 2024 Nov 19;20(11):e1012729. doi: 10.1371/journal.ppat.1012729 (PMC11642994; doi:10.1371/journal.ppat.1012729)
Supplement: S3 Table — (DOCX) [file ppat.1012729.s008.docx]

**S3 Table**

| Name of Peptides | Accession Number | Peptide Km (µM) in the presence of EhAGCK1 | Peptide Km (µM) in the presence of EhAGCK2 |
| --- | --- | --- | --- |
| Coactosin | EHI_168340 | 1.869$\pm$1.24 µM | 4.106$\pm$0.91 µM |
| Unconventional Myosin IB | EHI_110810 | 3.629$\pm$2.58 µM | 1.338$\pm$0.57 µM |
| RhoGEF-1 | EHI_159500 | 4.424$\pm$2.75 µM | 0.2706$\pm$0.17 µM |
| Actophorin | EHI_197480 | 2.825$\pm$1.69 µM | 0.2584$\pm$0.09 µM |
| RhoGEF | EHI_008090 | 2.078$\pm$0.80 µM | 0.1687$\pm$0.10 µM |
| Filamin-A interacting protein | EHI_025370 | 3.281$\pm$1.19 µM | 0.1475$\pm$0.71 µM |
| HEAT repeat domain-containing protein | EHI_050150 | 3.619$\pm$1.34 µM | 0.2624$\pm$0.20 µM |
| Hypothetical protein | EHI_025430 | 2.558$\pm$0.99 µM | 0.1970$\pm$0.08 µM |
